# Biomarker-Guided Non-Adaptive Trial Designs in Phase II and Phase III: A Methodological Review

**DOI:** 10.3390/jpm7010001

**Published:** 2017-01-25

**Authors:** Miranta Antoniou, Ruwanthi Kolamunnage-Dona, Andrea L. Jorgensen

**Affiliations:** 1 MRC North West Hub for Trials Methodology Research, Liverpool L69 3GL, UK; Ruwanthi.Kolamunnage-Dona@liverpool.ac.uk (R.K.-D.); A.L.Jorgensen@liverpool.ac.uk (A.L.J.); 2 Department of Biostatistics, Institute of Translational Medicine, University of Liverpool, Liverpool L69 3GL, UK

**Keywords:** biomarker-guided trial design, clinical research design, phase II, phase III, personalized medicine, predictive biomarker, prognostic biomarker, non-adaptive trial designs, clinical trials methodology, sample size

## Abstract

Biomarker-guided treatment is a rapidly developing area of medicine, where treatment choice is personalised according to one or more of an individual’s biomarker measurements. A number of biomarker-guided trial designs have been proposed in the past decade, including both adaptive and non-adaptive trial designs which test the effectiveness of a biomarker-guided approach to treatment with the aim of improving patient health. A better understanding of them is needed as challenges occur both in terms of trial design and analysis. We have undertaken a comprehensive literature review based on an in-depth search strategy with a view to providing the research community with clarity in definition, methodology and terminology of the various biomarker-guided trial designs (both adaptive and non-adaptive designs) from a total of 211 included papers. In the present paper, we focus on non-adaptive biomarker-guided trial designs for which we have identified five distinct main types mentioned in 100 papers. We have graphically displayed each non-adaptive trial design and provided an in-depth overview of their key characteristics. Substantial variability has been observed in terms of how trial designs are described and particularly in the terminology used by different authors. Our comprehensive review provides guidance for those designing biomarker-guided trials.

## 1. Introduction

The rapidly developing field of ‘personalized medicine’ [1], also known as ‘individualized medicine’, ‘stratified medicine’, or ‘precision medicine’ is allowing scientists to treat patients by providing them with a specific regimen according to their individual demographic, genomic or biological characteristics. The latter two aforementioned characteristics are collectively known as biomarkers [2]. The terms ‘personalized medicine’ and ‘individualized medicine’ often create confusion in literature, as in reality, the objective of this approach is to identify demographic- or biomarker-defined subgroups. Thus, as it still remains a population and not an individualized approach, the terms ‘stratified’ or ‘precision’ medicine are often considered to be more accurate. The National Institutes of Health Biomarkers Definitions Working Group [3] defined a biomarker to be “a characteristic that is objectively measured and evaluated as an indicator of normal biological processes, pathogenic processes, or pharmacologic responses to a therapeutic intervention” [1,4,5,6,7]. Biomarkers related to clinical outcome which are measured before treatment commences can be classified as either prognostic or predictive biomarkers. Prognostic biomarkers provide information regarding the likely progression of a disease without taking into account any specific treatment, whilst predictive biomarkers provide information about the patient’s outcome given a certain treatment, i.e., their likely response to the treatment [4,7,8,9,10,11,12,13,14,15,16,17,18,19,20,21,22,23,24,25,26,27,28,29,30,31,32,33,34]. Prior to utilizing a patient’s biomarker information in clinical practice, it is necessary that they have been robustly tested in terms of analytical validity (the results of testing a specific biomarker or biomarkers can be trusted), clinical validity (the results obtained from the test correlates with important clinical information) and clinical utility (the test will be useful in ameliorating patients’ health) [9,13,19,25]. 

A number of phase II and phase III trial designs have been proposed for testing the clinical utility of prognostic biomarkers. Due to the large amount of literature in this field, we have split our review into two broad categories, i.e., the biomarker-guided non-adaptive trial designs which are presented in the current study and the biomarker-guided adaptive trial designs. The latter are extensively discussed in our published paper “Biomarker-Guided Adaptive Trial Designs in Phase II and Phase III: a Methodological Review”, Antoniou et al., 2016 [35]. 

In this review we aim to communicate the different non-adaptive biomarker-guided trial designs, which can be either randomized or non-randomized designs (e.g., single-arm designs), proposed in the literature so far and to report on the potential advantages and weaknesses of each. Although not included in the paper by Antoniou et al., 2016 [35] which describes and discusses adaptive designs, some designs discussed in the current paper, although not adaptive in the traditional sense, they own an adaptive element.

## 2. Methods and Findings

**Figure 1:**
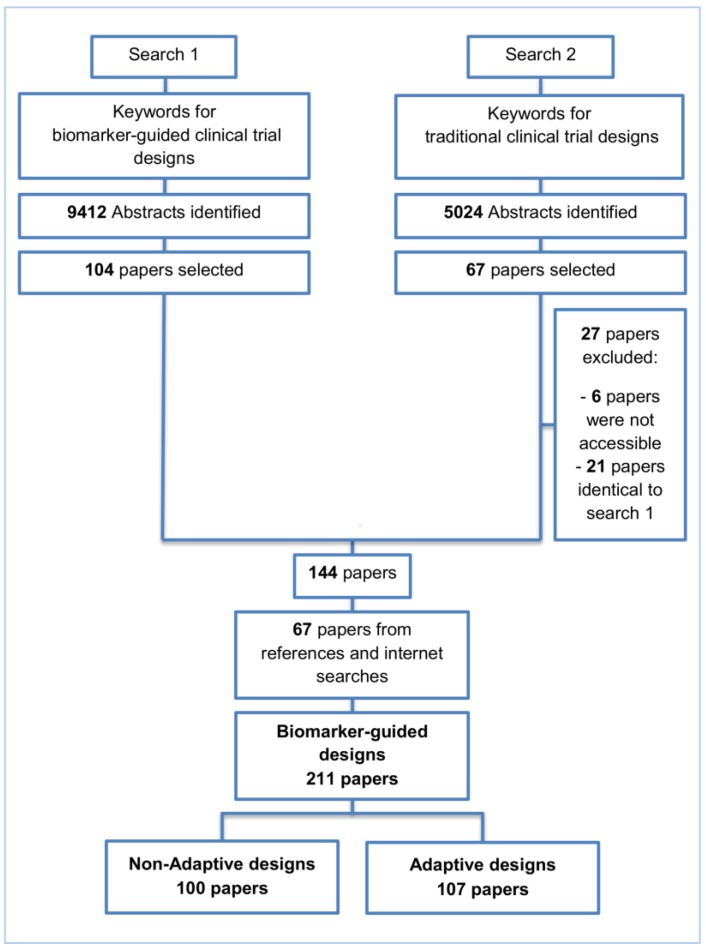
Flow diagram of the review process. From our search strategy a total number of 211 papers have been identified giving information regarding not only the biomarker-guided designs but also general information about personalized medicine and biomarkers. Before arriving at 211 papers, books, web pages for actual trials and papers published before 2005 were excluded. The 211 papers are split into two overlapping sets of 100 and 107 papers. The total of 207 is less than 211 due to overlap of papers, and also due to the fact that some articles referring to general information about personalized medicine and biomarkers and articles which do not provide further information on each broad of biomarker-guided designs were excluded. The 107 papers for biomarker-guided adaptive trial designs were reviewed in our published paper Antoniou et al. (2016) [35].

We undertook a search of the MEDLINE (Ovid) database, restricted to published papers in the English language within the previous ten years aiming to identify articles which describe and discuss biomarker-guided trial designs. Traditional trial designs, i.e., designs which do not incorporate biomarkers aiming to aid in making treatment decisions (we will refer to as ‘traditional’ trial designs) are part of our literature review search strategy in order to help us identify and distinguish any potential reference to biomarker-guided designs, as the finding of the appropriate keywords in Medline database for biomarker-guided designs was challenging. Furthermore, the restriction of published papers within the past decade was made not only because of the large amount of literature in this field, but also for the identification of the most recent trial designs. Two separate strategies as illustrated in Figure 1 were used to identify relevant articles, and the keywords utilized in the search are presented in S1 Keywords. Our initial search resulted in 9412 and 5024 relevant titles for biomarker-guided clinical trial designs and traditional trial designs, respectively. From the 9412 papers, 104 articles were included based on their title and abstract. From the 5024 papers, 40 articles were included based on their title and abstract and after removing inaccessible articles or those already identified in the search for biomarker-guided trial designs. An additional 67 eligible papers were identified from searching both the reference list of included articles and the internet (the internet searches were performed using the same keywords as those for the Ovid strategy), making a total of 211 included papers. Of these 211 included papers, biomarker-guided non-adaptive trial designs were referred to in 100 papers; 107 papers for biomarker-guided adaptive trial designs were reviewed in our published paper Antoniou et al., 2016 [35]. In the total number of 211 papers, some papers are referred to both adaptive and non-adaptive designs. Articles from references and internet searches which did not provide further information on each broad category of biomarker-guided designs were not included. Cited books, web pages for actual trials and papers published before 2005 are also not included in these numbers. For each included paper, the following details were extracted: definition of the trial design(s) referred to in the paper, how patients were screened and/or randomized based on their biomarker status, treatment groups randomized to, as well as other key information relating to the trial design and methodology, including advantages and limitations. Where reference was made in the included papers to an actual trial which had adopted a particular biomarker-guided non-adaptive trial design, the clinical field with which the trial was associated was also recorded. However, a review of all implementations of the different trial designs in practice is beyond the scope of this paper; however, and is an area for potential future work. Therefore, it is important to highlight that even where no evidence of the implementation of a particular design was found in the papers included in our review, the design may well be currently in use in ongoing trials.

**Figure 2:**
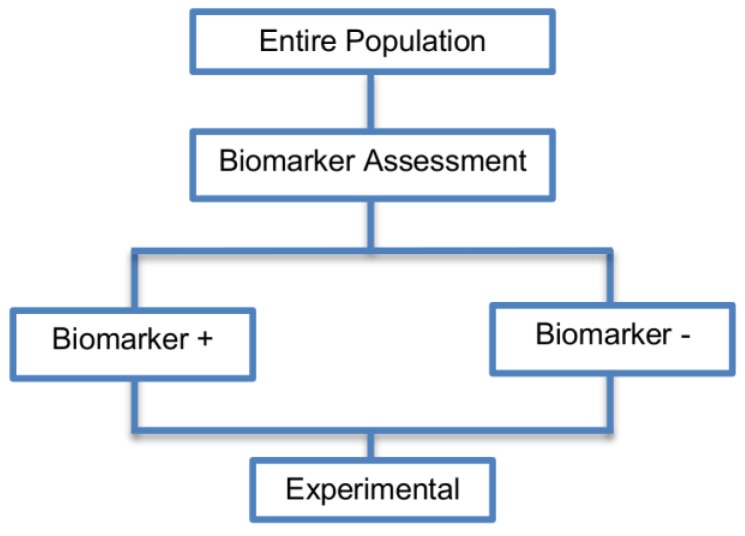
Single arm designs.

**Figure 3:**
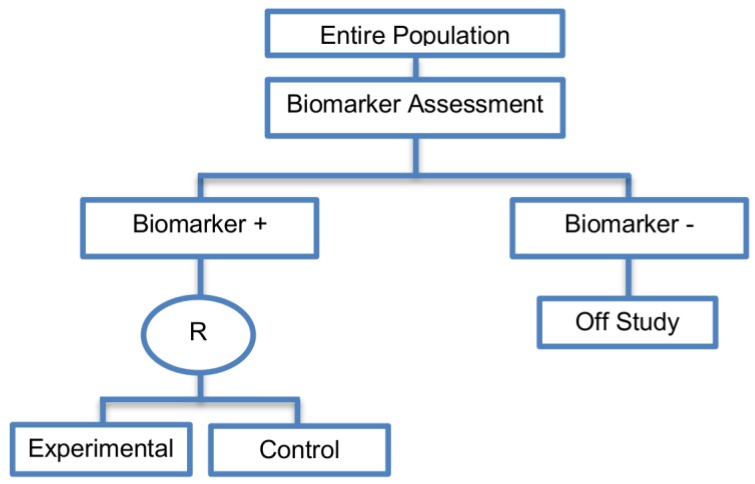
Enrichment designs. “R” refers to randomization of patients.

**Figure 4:**
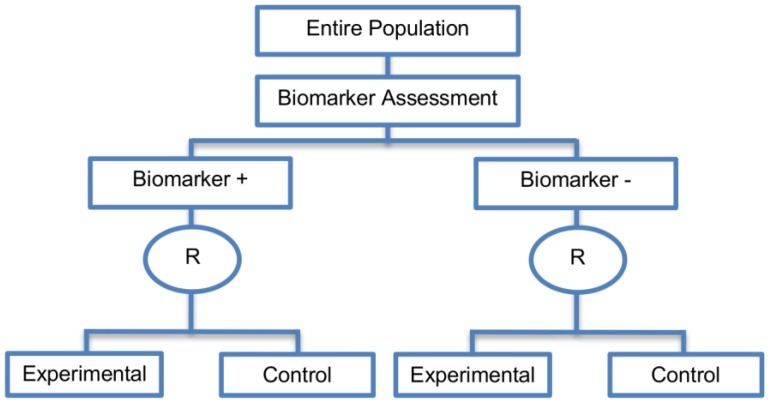
Marker Stratified designs. “R” refers to randomization of patients.

**Figure 5:**
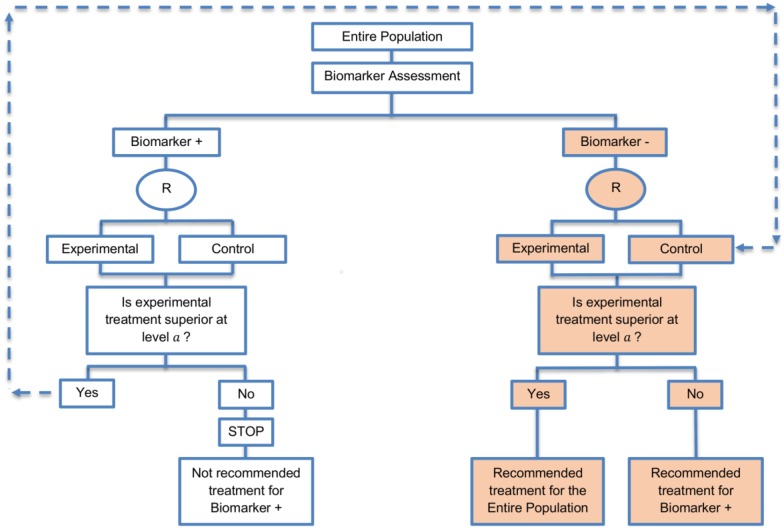
Sequential Subgroup-Specific design. “R” refers to randomization of patients. Uncolored boxes are referred to the first stage of the trial and colored boxes are referred to the second stage of the trial.

**Figure 6:**
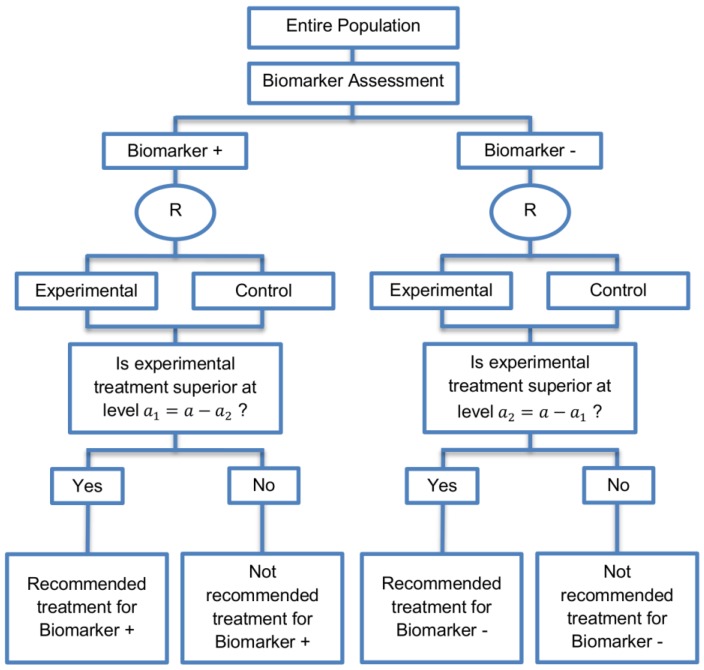
Parallel Subgroup-Specific design. “R” refers to randomization of patients.

**Figure 7:**
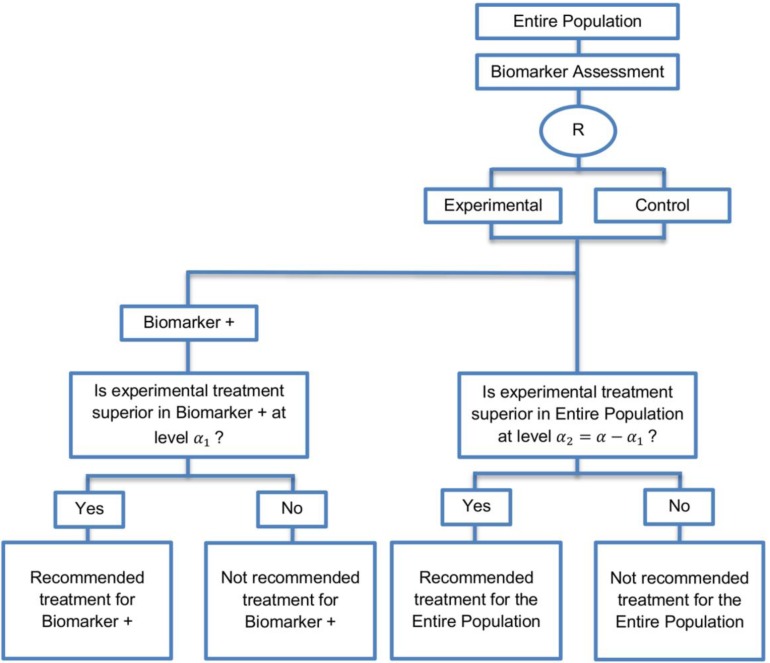
Biomarker-positive and overall strategies with parallel assessment. “R” refers to randomization of patients.

**Figure 8:**
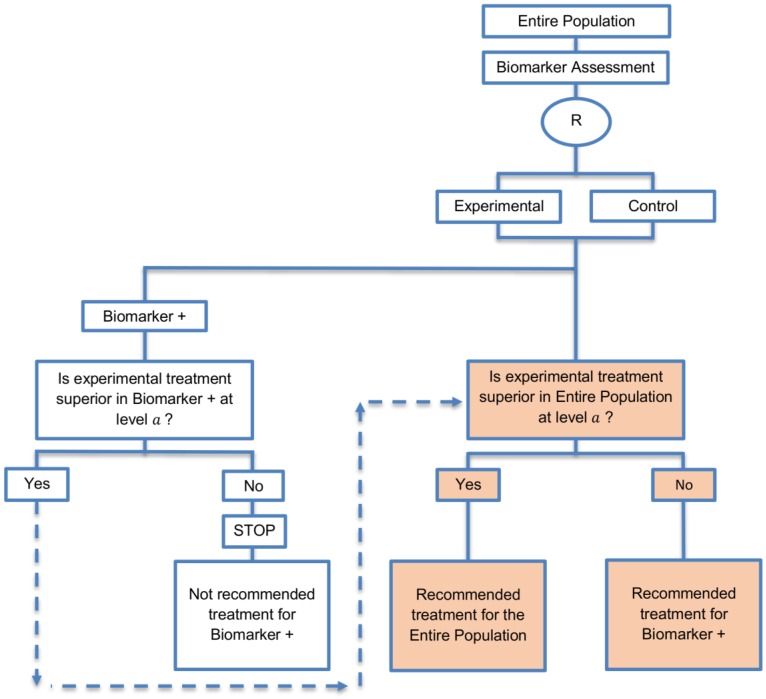
Biomarker-positive and overall strategies with sequential assessment. “R” refers to randomization of patients. Uncolored boxes are referred to the first stage of the trial and colored boxes are referred to the second stage of the trial.

**Figure 9:**
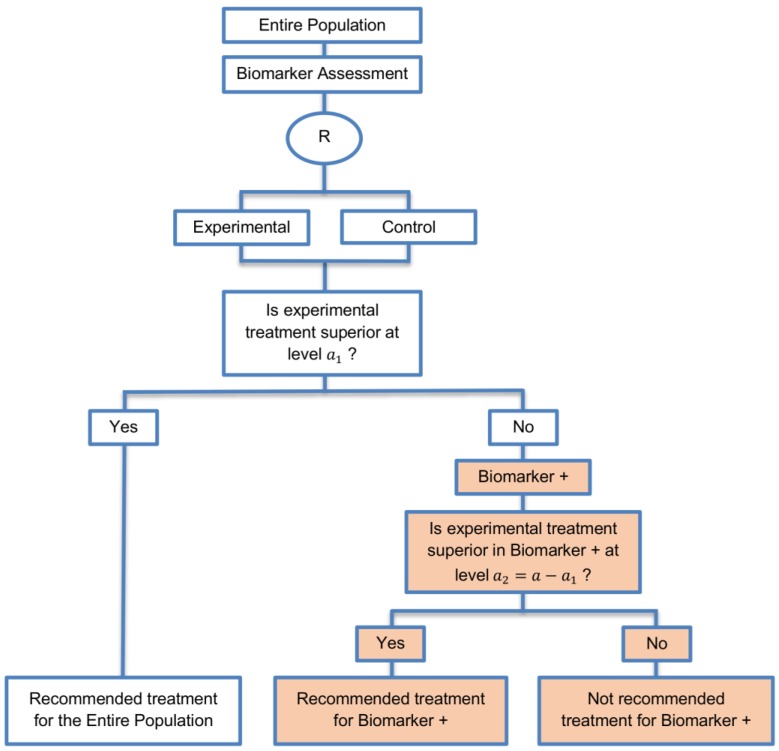
Biomarker-positive and overall strategies with fall-back analysis. “R” refers to randomization of patients. Uncolored boxes are referred to the first stage of the trial and colored boxes are referred to the second stage of the trial.

**Figure 10:**
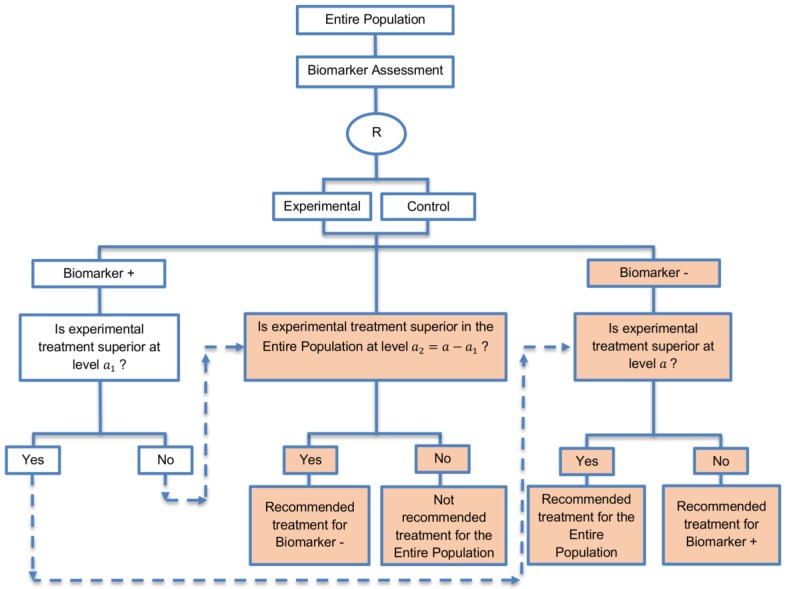
Marker Sequential test design (MaST). “R” refers to randomization of patients. Uncolored boxes are referred to the first stage of the trial and colored boxes are referred to the second stage of the trial.

**Figure 11:**
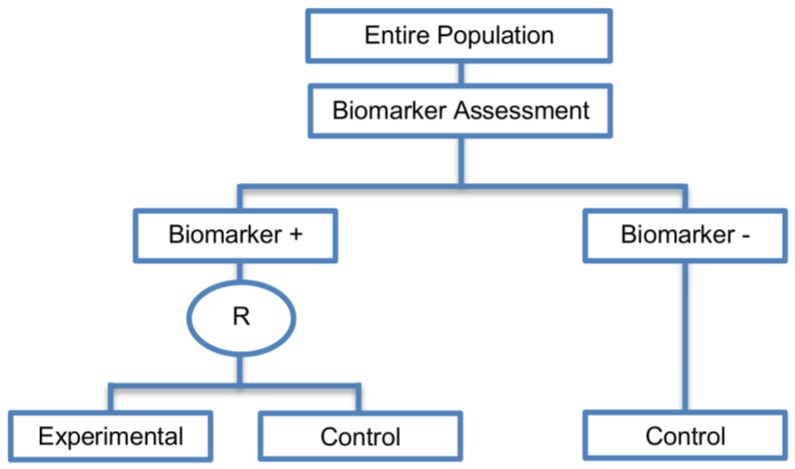
Hybrid design. “R” refers to randomization of patients.

**Figure 12:**
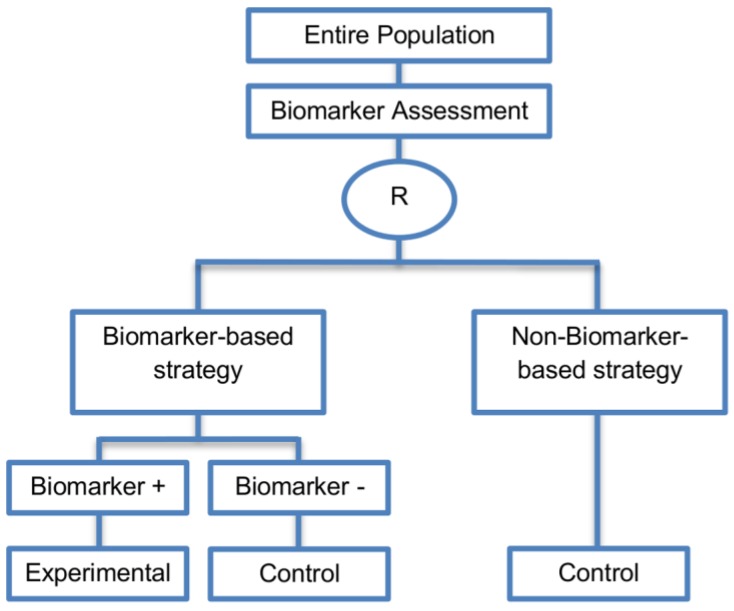
Biomarker-strategy design with biomarker assessment in the control arm. “R” refers to randomization of patients.

**Figure 13:**
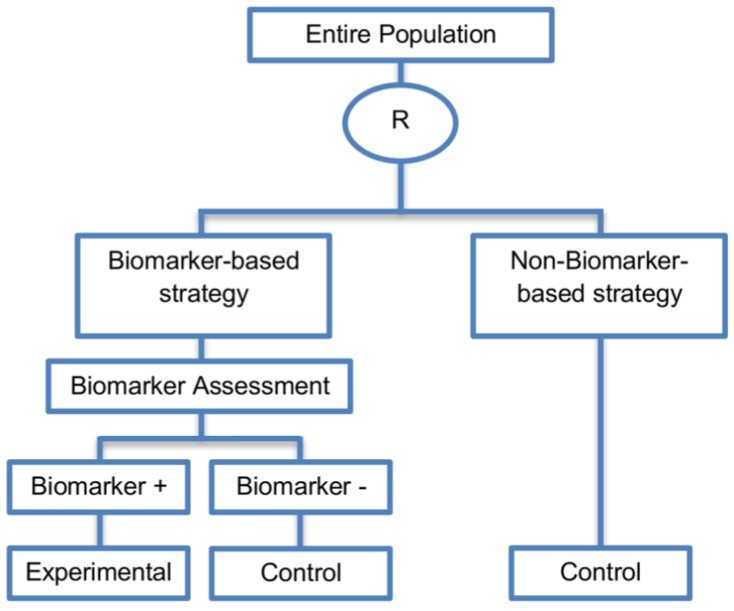
Biomarker-strategy design without biomarker assessment in the control arm. “R” refers to randomization of patients.

**Figure 14:**
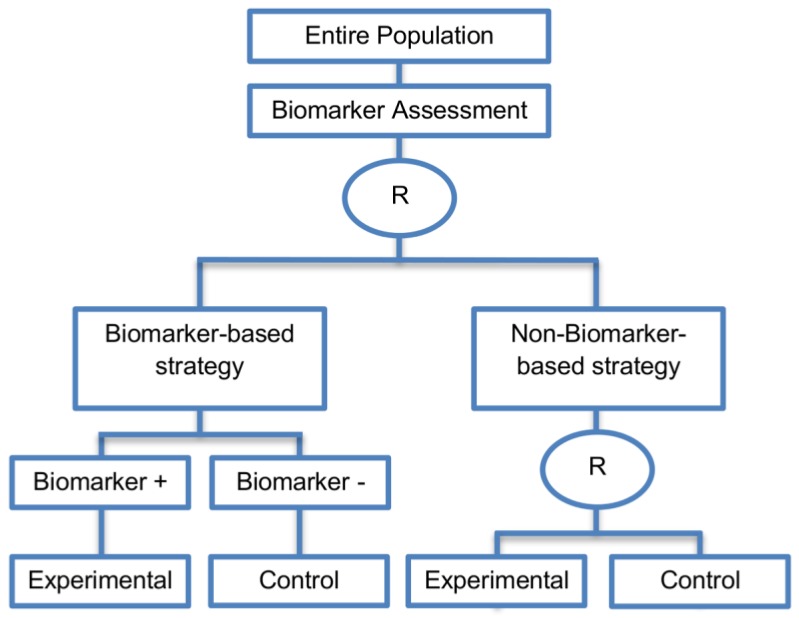
Biomarker-strategy design with treatment randomization in the control arm. “R” refers to randomization of patients.

**Figure 15:**
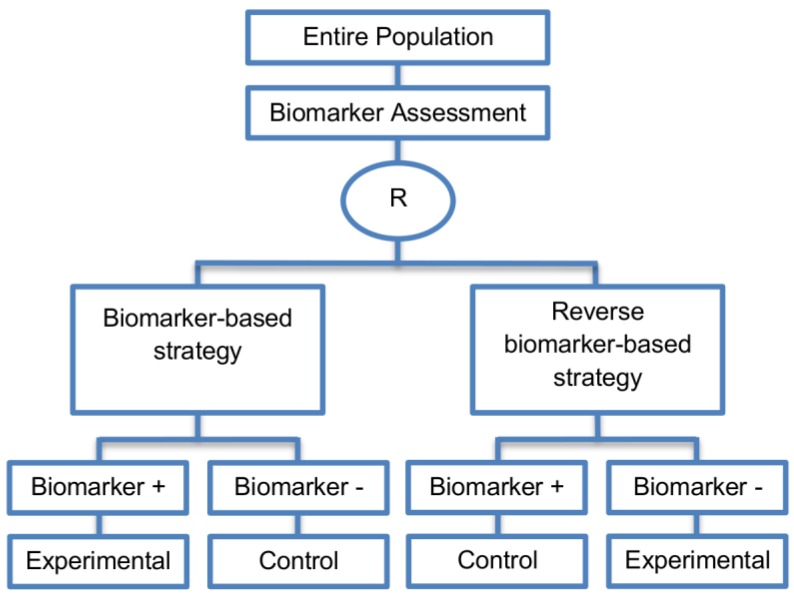
Reverse Marker-Based strategy design. “R” refers to randomization of patients.

**Figure 16:**
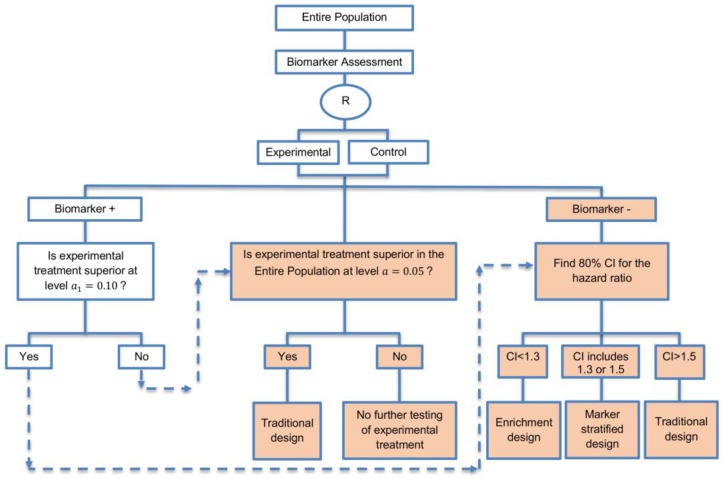
Randomized Phase II trial design with biomarkers. “R” refers to randomization of patients. CI refers to the confidence interval. Uncolored boxes are referred to the first stage of the trial and colored boxes are referred to the second stage of the trial.

In our review, we identified five main biomarker-guided non-adaptive trial designs namely: (i) single-arm designs; (ii) enrichment designs; (iii) randomize-all designs; (iv) biomarker-strategy designs and (v) other designs. Within each main design several subtypes and extensions were also identified. Graphical representations of the main designs and subtypes are given in Figure 2, Figure 3, Figure 4, Figure 5, Figure 6, Figure 7, Figure 8, Figure 9, Figure 10, Figure 11, Figure 12, Figure 13, Figure 14, Figure 15 and Figure 16. Graphical representations of the extensions are given in Figures S1-S4 included in File S1-S4. The characteristics and methodology of the main design types and subtypes are discussed below and are summarized in Table 1, whilst information on the extensions are discussed in File S1-S4. Furthermore, sample size formulae for each biomarker-guided design are provided in Table 2.

### 2.1. Single Arm Designs

Single arm designs were referred to in seven papers (7%). In the context of biomarkers, these designs (Phase II designs) include the whole study population to which the same experimental treatment is prescribed, without taking into consideration biomarker status. 

**Design:** In this design all patients are prescribed the experimental treatment and there is no comparison with a control treatment. These trial designs aid in the identification of association between biomarker status and the efficacy or safety of the experimental treatment. An illustration of this approach is shown in Figure 2.

**Utility:** These designs can be useful for the initial identification and/or validation of a biomarker and their aim is not to estimate the treatment effect in a definitive way but to identify whether the biomarker is sufficiently promising to proceed to a definitive Phase III biomarker-guided randomized controlled trial.

**Methodology:** In single arm designs first we assess the biomarker status of patients and then as all patients will be treated the same way we could compare the outcome of the biomarker-positive subgroup with the outcome of biomarker-negative subgroup. According to Tajik et al., 2012 [117], in terms of the required sample size, a standard formula can be used, however one should take into consideration the multiple testing issue that arise due to the exploration of several prognostic biomarkers (e.g., Bonferroni adjustment or normal exact method to protect against type I error a for multiple tests are often considered [118]). Further information can be found in the paper of Zaslavasky and Scott, 2012 [118] who studied the sample size estimation in single arm clinical trials with multiple testing under frequentist and Bayesian framework.

**Statistical considerations:** The single arm approach can be considered as a simple statistical design as there is no need for randomization. However one limitation of this strategy is that there is no distinction between prognostic and predictive biomarkers i.e., as patients are not randomized to experimental and control treatment groups, it is not possible to determine whether an observed effect is attributable to the natural disease progression or to the treatment. Consequently, this study designs are unable to show the benefit of a biomarker with regard to the best choice of treatment.

### 2.2. Enrichment Designs

Enrichment designs are described in 71 papers (71%), either in Phase II or Phase III clinical trials, and involve randomizing only the biomarker-positive patients and comparing the experimental treatment versus the standard treatment only in this particular biomarker-defined subgroup.

**Design:** Figure 3 graphically represents the trial design. First, the entire population is screened in order to identify the biomarker status of each patient. Next, the random assignment of individuals to different treatment arms is restricted only to the biomarker-positive subgroup. More precisely, biomarker-negative patients are excluded from the study and consequently, the assessment of the effectiveness of the experimental treatment is limited to the biomarker-positive subgroup. Thus, other patients apart from the biomarker-positive subpopulation can receive only the standard treatment (i.e., control treatment), but they are not included in the investigation during the trial design. The biomarker in this design is referred to as either the ‘selection’ or ‘enrichment’ biomarker.

**Utility:** Enrichment designs are useful for clinical trials aiming to test the treatment effect in a specific biomarker-defined subpopulation where there is evidence to suggest that effectiveness is limited to those within that subgroup, but the candidate biomarker still requires prospective validation. This design is recommended when both the cut-off point for determination of biomarker status of patients and the analytical validity of the biomarker have been well established. A rapid turnaround time for assessing the biomarker status of a patient is also needed to avoid any delay in treatment initiation. This strategy is particularly useful where it is unethical to randomize the biomarker-negative population into different treatment arms, for example where there is prior evidence that the experimental treatment is not beneficial for biomarker-negative individuals, or is likely to cause them harm. However, when it remains unclear whether or not biomarker-negative individuals will benefit from the novel treatment, the enrichment design is not appropriate and alternative designs, which also assess effectiveness in the biomarker-negative individuals, should be considered (e.g., randomize-all designs). 

**Methodology:** An online tool has been developed by Zhao and Simon [19,28,53,57,60] that allows sample size planning for the enrichment design both for binary and time-to-event (survival) outcomes, and is available at http://brb.nci.nih.gov/brb/samplesize/td.html [113]. For the purpose of estimating the sample size in the case of a survival outcome, data are simulated based on a marker stratified design (see next section for further information) in which both biomarker-positive and biomarker-negative subgroups are investigated in the study and formulae for the enrichment design described in the paper of Rubinstein et al., 1981 [110] are used. Furthermore, an exponential distribution of survival for the experimental and control treatment groups within both the biomarker-positive and biomarker-negative subpopulations is assumed. More precisely, Rubinstein et al.provide the formula of the expected number of events per treatment group allowing to include exponential loss to follow-up given the following assumptions: (i) patients enter the trial according to a Poisson process and patient entry times will be independent and identically distributed uniformly over [0,T] where T denotes the accrual time. Consequently, given the total number of patients N, the times from entry to the end of the trial will be independent and identically distributed uniformly over [τ,T+τ], where τ denotes the follow-up time and T+τ the total duration of the study and (ii) 1:1 randomization between experimental and control treatment group is considered. The expected number of events per treatment arm according to Rubinstein et al. is given by

(1) $$E\left(D_{i,enrichment}\right)=\frac{nT\lambda_{i}}{2\left(\lambda_{i}+\varphi_{i}\right)}\left\{1-\frac{e^{-\left(\lambda_{i}+\varphi_{i}\right)t}}{\left(\lambda_{i}+\varphi_{i}\right)T}\left[1-e^{-\left(\lambda_{i}+\varphi_{i}\right)T}\right]\right\},$$

where i corresponds to either the experimental or the control treatment group, λ corresponds to the event hazard rate, φ is the loss to follow-up rate and patients enter the trial according to a Poisson process with rate n per year over the accrual period of T years. However, the required total number of events in the two treatment groups (experimental and control treatment group) is given by

(2) $$D_{enrichment}=4{\left[\frac{\left(z_{\alpha /2}+z_{\beta }\right)}{{\log\theta }_{1}}\right]}^{2},$$

where $\theta_{1}$ denotes the assumed hazard ratio between the two treatment groups (control vs. experimental) in the biomarker-positive subset and the constants $z_{\alpha /2},\;z_{\beta }$ denote the upper α/2- and upper β-points respectively of a standard normal distribution where α and β denote the assumed type I error and type II error respectively. Freidlin et al., 2010 [61] provided the aforementioned formula assuming that all random assignments use 1:1 randomization. As in a traditional randomized controlled trial, if the randomization is not equal, i.e., the ratio of allocation to treatment and control is R:1 rather than 1:1, the aforementioned formula for the required total number of events $D_{enrichment}$ which assumes 1:1 randomization can be multiplied by ${\left(R+1\right)}^{2}/4R$ [119]. Consequently, the “4” in the formula of $D_{enrichment}$ becomes ${\left(R+1\right)}^{2}/R$ and the corresponding formula for the total number of events becomes

(3) $$D_{enrichment}=\frac{{\left(R+1\right)}^{2}}{R}{\left[\frac{\left(z_{\alpha /2}+z_{\beta }\right)}{{\log\theta }_{1}}\right]}^{2}.$$

In a survival study, the calculation of the total sample size in terms of number of patients required in the two treatment groups (experimental and control treatment group) to be enrolled in order to yield the aforementioned total number of events depends on the probability of event over the duration of the study [120]. Consequently, the actual number of patients required in a survival study can be given by

(4) $$N_{enrichment}=\frac{D_{enrichment}}{\mathrm{Pr}\left(event\right)},$$

where Pr(event) is the probability of observing an event in the two treatment groups in the study and $D_{enrichment}$ is the required total number of events. Pr(event) in a survival study can be given by

(5) $$\mathrm{Pr}\left(event\right)=\pi_{A}Pr_{A}\left(event\right)+\pi_{B}Pr_{B}\left(event\right),$$

where

(6) $$\pi_{A}=\frac{R}{R+1}\;\mathrm{and}\;\pi_{B}=\frac{1}{R+1},$$

are the proportions of patients who are randomized to experimental and control treatment group respectively and $Pr_{A}\left(event\right)$ and $Pr_{B}\left(event\right)$ are the probability of events in experimental and control arm respectively [121]. Freedman, 1982 [122] provided an approximation of the probability of event for each treatment group assuming equal follow-up for all patients and thus simultaneous accrual for all patients whereas Schoenfeld, 1983 [123] provided a more exact approximation of the expected event rate as compared to Freedman’s approximation. More precisely, according to Freedman’s idea,

(7) $$Pr_{i}\left(event\right)\approx 1-S_{i}\left(\tau \right)$$

and according to Schoenfeld’s idea,

(8) $$Pr_{i}\left(event\right)\approx 1-\left\{S_{i}\left(\tau \right)+4S_{i}\left(T/2+\tau \right)+S_{i}\left(T+\tau \right)\right\}/6,$$

where i denotes the corresponding treatment group (either experimental or control), τ denotes the follow-up time and T the accrual period, T/2+τ denotes the median follow-up time and T+τ denotes the total duration of the study. Another approximation of the probability of event could be

(9) $$Pr_{i}\left(event\right)\approx 1-S_{i}\left(T/2+\tau \right)$$

considering that the survival probability can be approximated as the probability that a patient survives past the median follow-up time (i.e., T/2+τ) [121].

The web-based interface is composed of two options. If the first option is chosen, the treatment effects for assay-negative and assay-positive patients must be specified in order to evaluate the relative efficiency of enrichment and untargeted design, i.e., marker stratified design (see next section for further information) in which apart from the biomarker-positive patients, biomarker-negative patients are also included; if the second option is chosen, it is possible to account for error in the assaying of the study population, thus, both the treatment effects for target-negative and target-positive patients must be specified as well as the assay’s sensitivity and specificity.

The sample size calculation using binary data is based on the formulas described by Simon and Maitournam [65,111,112] and again the two options offered when assuming a time-to-event outcome are available, i.e., options both with and without accounting for error in assaying the study population the biomarker status. When binary outcome is assumed and the allocation ratio is 1:1, the sample size of randomized patients required in each treatment arm (experimental and control) can be given as

(10) $$N_{enrichment/arm}=2{\bar{p}}_{Q}\left(1-{\bar{p}}_{Q}\right){\left[\frac{\left(z_{\alpha /2}+z_{\beta }\right)}{\left(p_{A}^{Q}-p_{B}\right)}\right]}^{2},$$

where $p_{A}^{Q}$ and $p_{B}$ are the response probabilities in the experimental and control groups respectively,

(11) $${\bar{p}}_{Q}=\frac{p_{A}^{Q}+p_{B}}{2}$$

and $z_{\alpha /2},\;z_{\beta }$ denote the upper α/2- and upper β-points respectively of a standard normal distribution where α and β denote the assumed type I error and type II error respectively. The response probability in the experimental group can be found by

(12) $$p_{A}^{Q}=p_{B}+\delta_{+},$$

where $\delta_{+}$ denotes the improvement in response probability for biomarker-positive patients. Consequently, the total sample size of randomized patients will be

(13) $$N_{enrichment}=2N_{enrichment/arm}$$

For continuous response endpoints the aforementioned formula $N_{enrichment/arm}$ changes to

(14) $$N_{enrichment/arm}=\frac{2\sigma^{2}{\left(z_{\alpha /2}+z_{\beta }\right)}^{2}}{{\left(\mu_{A+}-\mu_{B+}\right)}^{2}},$$

where $\sigma^{2}$ denotes the anticipated common variance, $\mu_{A+}$ and $\mu_{B+}$ the mean responses for biomarker-positive patients in the experimental and control treatment arm respectively. These formulae are the standard formulae used for a standard randomized trial.

In addition, if we want to account for error in the assaying of the study population, the number of patients to be randomized in each arm of the enrichment trial when using continuous response endpoints can be given by the following formula

(15) $$N_{enrichment/arm}=2\sigma^{2}{\left(z_{\alpha /2}+z_{\beta }\right)}^{2}{\left\{\lambda_{1}\left[\left(1-\omega \right)\;\zeta +\omega \right]\right\}}^{-2}$$

where  ω measures the accuracy of the assay and corresponds to the PPV (positive predictive value of the assay, i.e., the proportion of patients who are assigned the biomarker-positive status according to the assay who are truly biomarker positive), $\lambda_{1}$ is the treatment effect in the biomarker-positive patients and $\zeta =\lambda_{0}/\lambda_{1}$ (where $\lambda_{0}$ is the treatment effect in the biomarker-negative patients) [55].

Simon and Maitournam [65,111,112] considered that apart from the number of patients to be randomized, the number of patients needed to be screened should be also reported. Thus, they stated that the expected number of patients to be screened in the enrichment design is $N_{enrichment}/k$ where k corresponds to the proportion of biomarker-positive patients. The online tool developed by Zhao and Simon provides both the number of patients to be screened and to be randomized.

**Statistical considerations:** Simon and Maitournam [65,111,112] undertook a simulation study, assuming a binary outcome, to compare power of the enrichment design with an untargeted design (i.e., marker stratified design, see next section for further information) in which all patients are randomized without measuring the biomarker. They concluded that the efficiency of the enrichment design relies both on the prevalence of the biomarker-positive patients and on the accuracy of the assay. Whilst in the situation where the assay cut-off point is not well established, there is a risk of severely compromising the power of the trial when using an enrichment design, if fewer than half of the entire study population are biomarker-positive and there is robust evidence that the experimental treatment does not benefit the biomarker-negative patients, the required number of randomized patients to allow sufficient power to detect a significant treatment effect is much smaller in the enrichment design than in the untargeted trial design. However, in the latter situation a greater number of individuals would need to be screened when using the enrichment design, and accruing the required number of biomarker positive patients could take a longer period of time. More precisely, Simon and Maitournam showed that an approximation of the ratio of the required number of patients to be randomized for the untargeted trial design as compared with the required number of patients randomized in the enrichment design when using binary outcome can be given by the following equation

(16) $$\frac{N_{stratified}}{N_{enrichment}}\approx \frac{1}{{\left[k+\left(1-k\right)\frac{\delta_{-}}{\delta_{+}}\right]}^{2}}={\left[\frac{\delta_{+}}{k\delta_{+}+\left(1-k\right)\delta_{-}}\right]}^{2},$$

where k denotes the proportion of biomarker-positive patients, $\delta_{-}$ and $\delta_{+}$ correspond to the treatment effectiveness (i.e., improvement in response probability) in biomarker-negative and biomarker-positive subgroups respectively. Consequently, in the situation where it is known that the novel treatment does not benefit the biomarker-negative patients at all, the ratio of the number of patients needed for randomization in the untargeted design relative to the number of patients required for the enrichment design is approximately

(17) $$\frac{N_{stratified}}{N_{enrichment}}\approx \frac{1}{k^{2}},$$

as $\delta_{-}=0$. For example, if half of patients are biomarker-positive (k=0.5) then a quarter of those needed to be randomized to the untargeted design trial would need to be randomized to the enrichment design trial. In cases where the novel treatment is half as effective in biomarker-negative patients as in the biomarker-positive patients (i.e., $\delta_{-}/\delta_{+}=1/2$), the aforementioned ratio changes to

(18) $$\frac{N_{stratified}}{N_{enrichment}}\approx \frac{4}{{\left(k+1\right)}^{2}}.$$

### 2.3. Randomize-All Designs

Randomize-all designs (also named as all-comers/untargeted/unselected/non-targeted/simple randomization designs) allow the inclusion of the entire population as eligible for randomization. Consequently, the whole study population who meet the eligibility criteria, is randomly assigned to the different treatment groups (experimental and control treatment group) regardless of biomarker status. This design allows assessment of treatment benefit for the entire population irrespective of biomarker status whilst at the same time allowing for treatment benefit to be tested in the two biomarker-defined subgroups separately.

Generally, they are useful when we are uncertain about the benefit of the experimental treatment in the overall population versus the biomarker-defined subgroups, the targeted treatment may benefit both biomarker-positive and biomarker-negative patients, the goal is to test the predictive ability of a biomarker, the assay reproducibility and accuracy is questionable, the turnaround time for biomarker assessment is long and the biomarker prevalence is high.

Randomize-all designs are composed of two main subtypes: the Marker-stratified designs and the Hybrid designs, which are discussed separately below.

#### 2.3.1. Marker Stratified Designs

These designs (prospective validation Phase III trials) were identified in 45 papers (45%) of our review.

**Design:** An illustration of the design is shown in Figure 4. Individuals are stratified into biomarker-positive and biomarker-negative subgroups according to the results of the biomarker assessment and then they are randomized either to the experimental or to the control treatment group. The biomarker status in the Marker-Stratified design acts as a stratification factor where stratification is used to ensure balance across treatment groups with regard to biomarkers. Only individuals with valid biomarker results enter the trial. Consequently, we have four treatment groups, i.e., biomarker-positive patients assigned to either the experimental treatment arm or the control treatment arm and biomarker-negative patients assigned to either the experimental treatment arm or the control treatment arm. Thus, we can assess the relationship between treatment effect and biomarker status.

**Utility:** When there is enough evidence that the experimental treatment is more effective in the positive biomarker-defined subgroup than in the negative biomarker-defined subgroup but there is no sufficient compelling data that the experimental treatment is of no benefit in biomarker-negative individuals, the marker stratified design can be used.

**Methodology:** Biomarker status is used to stratify the randomization, rather than to restrict eligibility. Marker-stratified designs can be conducted using two different testing plans; the so-called marker-by-treatment interaction with separate tests and marker-by-treatment interaction with interaction test. Both of these approaches involve conducting two independent clinical trials.

Marker-by-treatment interaction using separate test was referred to in 15 papers (15%) of our review [4,11,12,15,29,42,45,53,57,60,80,82,84,87,88] and is also referred to as ‘separate randomization design’ and ‘separate by treatment interaction design’. This analysis plan is based on separate superiority tests in each biomarker-defined subgroup in order to detect the treatment efficacy in each subset. Two examples of actual trials which use this testing plan are the following: National Cancer Institute (NCI)-sponsored North Central Cancer Treatment Group Study N0975 [29] and the MARVEL trial [29].

The ‘marker-by-treatment interaction design using separate tests’ is a testing plan which determines whether the novel treatment is superior to the control treatment separately within each biomarker-defined subgroup. Consequently, the hypothesis to be tested, the calculation of the number of patients required for the trial, the estimation of the statistical power of the design and the randomization procedure of patients to different treatments are independent among the different subgroups [12]. The sample size of the trial should be calculated in such a way so as to yield adequate statistical power when testing whether the experimental treatment is superior to the control treatment separately in the two biomarker-defined subgroups. Hence, this approach is not widely used due to the required large sample size as essentially two separate trials are being conducted. Another limitation of this approach is that when multiple biomarker-defined subsets and treatments are to be investigated, it is difficult to implement in practice.

The ‘marker-by-treatment interaction using interaction test’ uses a test for interaction between the biomarker status and treatment assignment and was identified in 12 papers (12%) of our review [4,12,15,42,53,57,60,82,84,87,88,94]. A marker stratified design which uses this testing plan is also referred to in the literature as an ‘interaction design’ or ‘genomic signature stratified design’. First, a formal statistical test for interaction between biomarker status and treatment assignment is undertaken. If this interaction is not significant, then the study is continued by testing the different treatments overall at a two-sided significance level of 0.05, otherwise, the treatments are compared within each biomarker-defined subpopulation at a two-sided 0.05 significance level (i.e., the same as in the marker-by-treatment interaction design using separate tests). The sample size for this second testing plan is calculated with reference to the treatment effect in the entire study population. Therefore, it might not provide sufficient power for detecting the treatment effect in each biomarker defined-subset individually. More precisely, if the sample size is calculated for the overall analysis and the proportion of the biomarker-defined subpopulation which responds to the novel treatment is very small, the statistical power for the subgroup analysis may be inadequate. In addition, when several biomarker-defined subpopulations and treatments are to be investigated, this strategy is not easy to be implemented.

For the case of binary outcomes, Eng, 2014 [92] provided the formula for the required sample size to power the biomarker-positive and biomarker-negative patients separately. It is assumed that Y is a binary variable which corresponds to a patient’s response to their randomly tailored treatment and $P\left(Y|Trt=i,\;M=j\right)=r_{ij}$ where i corresponds to either the experimental or control treatment and j corresponds to either the biomarker-positive patients or the biomarker-negative patients. Hence,

(19) $$r_{ij}=\beta_{0}+\beta_{A}I\left(Trt=A\right)+\beta_{+}I\left(M=M^{+}\right)+\beta_{I}I\left(Trt=A,\;M=M^{+}\right),$$

where $\beta_{0}$ denotes a baseline effect, $\beta_{A}$ denotes the added effect of the experimental treatment, $\beta_{+}$ denotes the biomarker-positive effect and $\beta_{I}$ denotes the nonadditive effect. Consequently, the proposed formula for the required sample size can be given by

(20) $$N_{stratified}=2{\left(z_{a}+z_{1-\beta }\right)}^{2}\left\{\frac{r_{A+}\left(1-r_{A+}\right)+r_{B+}\left(1-r_{B+}\right)}{{\left(\beta_{A}+\beta_{I}\right)}^{2}}+\frac{r_{A-}\left(1-r_{A-}\right)+r_{B-}\left(1-r_{B-}\right)}{{\left(\beta_{A}\right)}^{2}}\right\},$$

where α correspond to the target level, 1−β corresponds to the power. Also, $r_{A+},\;r_{B+}$ are the assumed response rates of biomarker-positive patients receiving the experimental and the control treatment respectively. Additionally, $r_{A-},\;r_{B-}$ are the assumed response rates of biomarker-negative patients receiving the experimental and the control treatment respectively.

Mandrekar and Sargent, 2009 [31] provide a formula to calculate the required number of events when the trial has a survival outcome with 1:1 randomization to treatment arms, i.e.,

(21) $$D_{stratified}=\frac{4{\left(z_{a/2}+z_{\beta }\right)}^{2}}{{\left[\log\left(\frac{m_{A+}}{m_{B+}}\right)\right]}^{2}}+\frac{4{\left(z_{a/2}+z_{\beta }\right)}^{2}}{{\left[\log\left(\frac{m_{A-}}{m_{B-}}\right)\right]}^{2}},$$

where $m_{A+},m_{A-},m_{B+},m_{B-}$, indicate the median overall survival for biomarker-positive and biomarker-negative patients receiving control and experimental treatment, respectively and

(22) $$\theta_{1}=\frac{m_{A+}}{m_{B+}}=HR_{biom^{+}},$$

(23) $$\theta_{2}=\frac{m_{A-}}{m_{B-}}=HR_{biom^{-}},$$

correspond to the hazard ratios of biomarker-positive and biomarker-negative subgroups and $z_{\alpha /2},\;z_{\beta }$ denote the upper α/2- and upper β-points respectively of a standard normal distribution where α and β denote the assumed type I error and type II error respectively. More precisely, the total number of events is the sum of the required number of events for the biomarker-negative and biomarker-positive subpopulation. Freidlin et al., 2010 [61] stated that the required number of events in order to compare the experimental to the control treatment among the biomarker-positive patients for detecting a given effect size in this biomarker-positive subpopulation is identical to the number of events needed by an enrichment design (i.e., $D_{enrichment}$).

Another potential formula for the required total number of events when 1:1 randomization to treatment arms is assumed is given by

(24) $$D_{stratified}=\frac{4{\left(z_{a/2}+z_{\beta }\right)}^{2}}{{\left[k\log\left(\theta_{1}\right)+\left(1-k\right)\log\left(\theta_{2}\right)\right]}^{2}}.$$

Although the formula proposed by Mandrekar and Sargent, 2009 [31] achieves a specific power (1−β) for each biomarker-defined subgroup separately, the aforementioned formula proposed in the book of Harrington, 2012 [114] aims to reach a power (1−β) for the overall population. According to Harrington, 2012 the required total number of patients to be entered to a stratified trial can be given by

(25) $$N_{stratified}=\frac{4{\left(z_{a/2}+z_{\beta }\right)}^{2}}{\left\{\frac{\left[kPr_{\left(+\right)}\left(event\right)\log\left(\theta_{1}\right)+\left(1-k\right)Pr_{\left(-\right)}\left(event\right)\log\left(\theta_{2}\right)\right]}{\sqrt{kPr_{\left(+\right)}\left(event\right)+\left(1-k\right)Pr_{\left(-\right)}\left(event\right)}}\right\}},$$

where $Pr_{\left(+\right)}\left(event\right)$, $Pr_{\left(-\right)}\left(event\right)$ are the probability of an event in biomarker-positive subset and biomarker-negative subset respectively. If we divide the required total number of events for the enrichment design by the aforementioned formula for the required total number of events for the stratified design, we can get the following approximation of the ratio

(26) $$\frac{D_{stratified}}{D_{enrichment}}=\frac{{\left[\log\left(\theta_{1}\right)\right]}^{2}}{{\left[k\log\left(\theta_{1}\right)+\left(1-k\right)\log\left(\theta_{2}\right)\right]}^{2}}=\frac{1}{{\left[k+\left(1-k\right)\frac{\log\left(\theta_{2}\right)}{\log\left(\theta_{1}\right)}\right]}^{2}}.$$

Further, Zhao and Simon [19,28,53,57,60] have developed an online tool for the calculation of sample size for biomarker stratified randomized designs with binary or time-to-event endpoints which is available online at the following web site http://brb.nci.nih.gov/brb/samplesize/sdpap.html [115]. More precisely, the sample size for both binary and time-to-event endpoints can be performed with three different analysis plans; A, B and C. Before choosing one of these analysis plans in the web site, for binary endpoints we need to specify the probability of treatment response in the control arm as well as the proportion of biomarker-positive patients. For survival endpoints, the hazard ratio of biomarker-positive patients versus the biomarker-negative control patients which corresponds to the hazard ratio of prognostic effect as well as the proportion of biomarker-positive patients must be specified.

Analysis plan A is performed when there is confidence that an overall treatment effect exists. It determines the sample size on the basis of first of all comparing the experimental treatment to the control treatment in the entire randomized population at a reduced two-sided significance level a<0.05. If the overall test is not significant, then the experimental treatment is compared to the control treatment in the biomarker-positive patients using the type I error a=0.05. Analysis Plan A is similar to the ‘Biomarker-positive and overall strategies design’ with fall-back analysis described later in this paper; the difference lies in this in terms of the significance levels they have used. In order for the sample size to be estimated, the anticipated overall effect estimate, reduced two-sided significance level and power for the overall test need to be specified.

Analysis plan B is performed when there is confidence that there is a treatment effect in the biomarker-positive subpopulation. It determines the sample size on the basis of first of all comparing the experimental treatment to the control treatment in the biomarker-positive subgroup at a two-sided significance level of a= 0.05 level. If the treatment effect is found to be significant at this 0.05 level, then treatment effect is evaluated in the biomarker-negative subgroup again at a two-sided significance level of 0.05 level. This analysis plan is identical to the ‘Sequential subgroup specific design’ described later in this paper. In order for the sample size to be estimated, apart from the fixed significance level set to 0.05, the anticipated effect estimate in the biomarker-positive subpopulation and power need to be specified.

Analysis plan C first tests whether there is a statistically significant interaction between treatment and biomarker [60] at a significance level a≤0.05. If the interaction is not significant, then the treatments are compared in the overall study population at a two-sided significance level 0.05. Otherwise, the treatments are compared within the two biomarker subgroups separately at a two-sided 0.05 significance level for each subgroup. Analysis Plan C follows either the ‘marker-by-treatment interaction process with interaction or the separate test process’ described above. In order for the sample size to be estimated, the anticipated treatment effect in the overall study population, the one-sided significance level for interaction test and the power for testing the treatment effect in the overall population need to be specified.

In marker stratified designs, three designs can be included which differ in terms of their statistical testing strategies, i.e., (i) Subgroup-specific designs (i.e., sequential subgroup-specific design, parallel subgroup-specific design); (ii) Biomarker-positive and overall strategies (i.e., biomarker-positive and overall strategies with parallel assessment, biomarker-positive and overall strategies with sequential assessment, biomarker-positive and overall strategies with fall-back analysis); (iii) Marker sequential test design (MaST) and they are discussed in the following sections.

**Statistical considerations:** Despite the fact that the marker stratified designs allow testing the treatment effect not only in the entire population but also in each biomarker-defined subpopulation, they might not be feasible when the prevalence of biomarker is low. Another limitation of such designs is that they might require a large sample size where several treatments and biomarkers are investigated in the study.

**Subgroup-Specific designs:** This strategy is an approach to analyze a biomarker-stratified trial. It is composed of two types; ‘Sequential Subgroup-Specific design’ and ‘Parallel Subgroup Specific design’. Both biomarker-positive and biomarker-negative subgroups can be tested in a sequential or in a parallel way. With the parallel way, we can assess simultaneously both biomarker-positive and biomarker-negative patients, whereas, with the sequential way we perform first the assessment of biomarker-positive patients and if the result is positive then we continue with the biomarker-negative patients.

**Sequential Subgroup-Specific design:** This approach was referred to in 11 papers (11%) of our review. Figure 5 graphically represents this approach.

**Design:** The sequential testing procedure uses the assumption that it is unlikely that the new treatment will be effective in the biomarker-negative patients unless it is effective in the biomarker-positive patients. First treatment effect is tested in the biomarker-positive subpopulation using the overall two-sided significance level α=0.05 (Type I error); if this test is significant then treatment effect is tested in the biomarker-negative subgroup using the same level of significance α.

**Utility:** Its use is recommended when there is compelling evidence that biomarker-positive individuals benefit more from the experimental treatment than the biomarker-negative patients. More precisely, it is appropriate when it is not expected for the novel treatment to be effective in biomarker-negative patients unless it is beneficial for the biomarker-positive patients.

**Methodology:** As this subgroup-specific design follows a sequential assessment and thus the design is composed of two stages, the sample size calculation is also staged. For binary outcome the required number of biomarker-positive patients is the same as for the enrichment design, i.e.,

(27) $$N_{Sequential\;subgroup-specific}^{+}=N_{enrichment}$$

As Simon, 2008 [60] stated, the total number of patients will be approximately

(28) $$N_{Sequential\;subgroup-specific}=\frac{N_{enrichment}}{k}$$

where k is the proportion of biomarker-positive patients and the number of biomarker-negative patients will be approximately

(29) $$N_{Sequential\;subgroup-specific}^{-}=\frac{\left(1-k\right)N_{enrichment}}{k}.$$

For the conduct of this design, it is important to ensure that there is also an adequate number of biomarker-negative patients for analysis purposes. For time-to-event outcomes, the required number of events for biomarker-positive patients is the same with the required number of events in the enrichment design, i.e.,

(30) $$D_{Sequential\;subgroup-specific}^{+}=D_{enrichment}.$$

At the time that there are $D_{enrichment}$ patients, the required number of events among biomarker-negative patients in terms of that among biomarker-positive patients $\left(D_{enrichment}\right)$ is given by

(31) $$D_{Sequential\;subgroup-specific}^{-}=D_{enrichment}\left(\frac{\lambda_{-}}{\lambda_{+}}\right)\left(\frac{1-k}{k}\right),$$

where $\lambda_{-}$, $\lambda_{+}$ are the event rates in biomarker-negative and biomarker-positive control subsets at the time when there are $D_{enrichment}$ events in the biomarker-positive subgroup [60].

The significance levels a can also be considered as one-sided significance levels in situations where our alternative hypothesis is not that there is just a treatment effect but that the treatment benefit in the experimental group is greater than that of the control group.

**Statistical considerations:** This strategy preserves the overall type I error rate a but requires a smaller number of positive patients as compared to the second type of subgroup-specific design, the so-called parallel subgroup-specific design (see below). Furthermore, it enables the identification of treatment efficacy in the biomarker-positive and biomarker-negative subpopulations separately. However, it yields low power when there is homogeneity of treatment effect across the different biomarker-defined subpopulations. Furthermore, in case that test for treatment effect among biomarker-negative patients is not statistically significant, an ‘exploratory’ analysis on the biomarker-negative subgroup might be considered.

**Parallel Subgroup-Specific design:** This design was identified in three papers (3%) of our review.

**Design:** Parallel subgroup-specific design (Phase III), also referred to as a Phase III Biomarker-Stratified design evaluates treatment effects separately in the positive biomarker-defined subgroup and in the negative biomarker-defined subgroup simultaneously. A graphical illustration of this strategy is given in Figure 6.

**Utility:** It is appropriate when the aim of the study is to give treatment recommendations for each biomarker-defined subgroup separately at the same time.

**Methodology:** In order to control the overall type I error rate of the design at the overall level of significance α (Type I error) it is required to allocate this overall α between the test for the biomarker-positive subgroup and the test for the biomarker-negative subgroup using the Bonferroni correction method [124] for multiple testing; e.g., if we choose the value of 0.025 for the global significance level α, then we could choose the values of $a_{1}=0.010$ and $a_{2}=0.015$ for testing the biomarker-negative and biomarker-positive subgroups respectively. This trial design is powered in such a way so as to detect the treatment effect in each biomarker-defined subgroup separately. A higher portion of the type I error rate can be given for the test within the biomarker-positive subgroup in order to maximize the power of the trial to identify the treatment effect in this subpopulation. However, even if there is a slight increase in the type I error probability spent on the test of one of the biomarker-defined subgroups, the power would probably not change much.

As in the sequential subgroup-specific design, the probability of rejecting either the null hypothesis of no treatment effect in the biomarker-positive subset or in the biomarker-negative effect under the global null hypothesis is less than or equal to the overall type I error rate a. Additionally, the probability of rejecting the null hypothesis of no treatment effect in the biomarker-negative subpopulation when the treatment benefit is only restricted to biomarker-positive patients is less than or equal to a. The significance levels a can be considered as one-sided or two-sided significance levels. 

**Statistical considerations:** With this approach, in case that the overall level of significance a is equal in both subgroup-specific designs, it is more difficult to achieve statistical significance in the biomarker-positive subgroup as compared to the sequential subgroup-specific design due to the allocation of the overall significance level between the two biomarker-defined subgroup tests.

**Biomarker-positive and overall strategies:** This design provides an alternative strategy to analysing a biomarker-stratified design. It is an indirect way of evaluating both biomarker and treatment by testing the treatment effect in the entire study population and in the biomarker-positive subgroup separately. Three approaches are included in the biomarker-positive and overall strategies; the parallel assessment, the sequential assessment and the fall-back design (see below).

Despite the fact that the biomarker-positive subgroup and overall strategy design allows the treatment effect to be tested in the biomarker-positive subpopulation and provides good statistical power when the treatment effect is homogeneous across subgroups, this design is usually considered problematic and its use is not often recommended. More precisely, a major concern is that when the benefit of the novel treatment is limited to the biomarker-positive patients, it is possible that the design might lead to a wrong recommendation of treatment for the biomarker-negative patients. This might happen because when there is no treatment effect in the biomarker-negative subgroup, there might be an observed effect in the entire population due to the potentially large effect in the biomarker-positive patients. This concern is particularly pronounced in the sequential version of the design, which first tests the biomarker-positive subgroup and then, if it is positive, it tests the overall population.

**Biomarker-positive and overall strategies with parallel assessment**: This approach was identified in eight papers (8%) of our review. Figure 7 graphically represents this strategy. In the parallel version, we test both the overall population and biomarker-positive subgroup simultaneously.

**Design:** In this approach the treatment effect is tested in both the entire study population and in the biomarker-positive patients while controlling the type I error by allocating the overall significance level α between the two tests. The significance level a can be considered as one-sided or two-sided.

**Utility:** The parallel version is recommended when the aim of the study is to assess the treatment effect in both the overall study population and in the biomarker-positive subgroup but not in the biomarker-negative subgroup.

**Methodology:** If there is significant confidence that the biomarker is predictive, the sample size estimation is aimed at having a sufficient number of biomarker-positive individuals to enable the treatment effect in the biomarker positive subgroup to be detected. On the other hand, if there is no confidence in the predictive value of the biomarker, the sample size estimation is aimed at having a sufficient number of patients to detect a treatment effect in the overall study population [14].

**Statistical considerations:** This design has the ability to control the probability of rejecting the null hypothesis of no treatment effect either in the biomarker-positive population or in the biomarker-negative population under the global null hypothesis of no treatment effect in the entire population at the overall significance level a. However, it cannot control the probability of rejecting the null hypothesis of no treatment effect in the biomarker-negative subset when the treatment benefit is restricted to biomarker-positive patients. Consequently, there is high risk of inappropriately recommending the experimental treatment for biomarker-negative patients.

When the experimental treatment is compared to the control treatment within the overall population and the overall treatment effect is significant, then the test has high statistical power. If we are testing only the biomarker-positive subgroup and the treatment effect in this subgroup is significant, the statistical power is again high. This prospective subset analysis plan is based on testing both the overall study population and the biomarker-positive subgroup using significance levels, which are chosen in such a way that the overall significance level is equal or less than a (type I error). An easy way is to split a in such a way that the significance level for the entire population and the significance level for the biomarker-positive subset equals to overall significance level a (typically a=0.05). For example, the SATURN trial (NCT00556712) [96] which employs a prospective subset strategy used the value of 0.03 as level of significance to test the treatment effect in the entire population and the value of 0.02 to test the treatment effect in the biomarker-positive subset; therefore, the overall level of significance was preserved at 0.05. The approach can be overly conservative as in the SATURN trial because of the correlation between the global and subgroup test. Other approaches [98,125,126,127,128] have been proposed for adjusting the level of significance of both tests in a more accurate and less conservative way.

**Biomarker-positive and overall strategies with sequential assessment:** This approach was referred to in 11 papers (11%) of our review. A graphical illustration of this approach is shown in Figure 8.

**Design:** In this sequential version of the biomarker-positive and overall strategies, we first test the biomarker-positive subgroup using the significance level α; if the test is significant, then we test the treatment effect in the overall population using the same α level. The significance levels a can be considered as one-sided or two-sided significance levels.

**Utility:** The sequential version might be useful in cases where the experimental treatment is expected to be effective in the overall study population. 

**Methodology:** As this design comprises two sequential stages, it follows that the sample size calculation should also be staged. At the first stage, the standard formula for a traditional randomized trial can be used for the biomarker-positive subgroup using the significance level α to estimate the treatment effect in that subset. More precisely, the formula used in the enrichment design for the required total number of events or the required number of patients can be used at the first stage of this design. At the second stage, the sample size must be adjusted in order to yield appropriate power for the entire population.

**Statistical considerations:** As in the parallel version of this designs, this strategy does not allow for identification of treatment efficacy in the biomarker-negative subgroup and despite the fact that it can control the overall type I error α it cannot control the probability of rejecting the null hypothesis of no treatment effect in the biomarker-negative subset when the treatment benefit is restricted to biomarker-positive patients. Consequently, for this design also there is high risk of inappropriately recommending the novel treatment for biomarker-negative patients.

**Biomarker-positive and overall strategies with fall-back analysis:** This strategy was identified in 15 papers (15%) of our review. It evaluates both the treatment effect in the overall study population and in the biomarker-positive subgroup sequentially. Figure 9 graphically represents this strategy.

**Design:** In the fall-back design, we first test the overall population using the reduced significance level $a_{1}$ and if the test is significant, we consider that the novel treatment is effective in the overall population; however, if the result is not significant then we test the treatment effect in the biomarker-positive subgroup using the level of significance $a_{2}=a-a_{1}$, where a is the overall significance level (Type I error rate). The significance levels a can be considered as one-sided or two-sided significance levels. The same analysis plan was used in the adaptive signature design which is further described in our methodological review regarding the biomarker-guided adaptive designs, Antoniou et al., 2016 [35]. More precisely, the difference between the adaptive signature design and the fall-back design is the following: in the adaptive signature design, in case that the first stage failures to show treatment effectiveness in the entire population, then the study population is divided in order to develop and validate a biomarker, using a split sample strategy, whereas in the biomarker-positive and overall strategies design with fall-back analysis the biomarker assessment is conducted at the beginning of the trial. However, both of the designs test at the first stage the entire population at the significance level $a_{1}$ and at the second stage the biomarker-positive patients at the significance level $a_{2}=a-a_{1}$.

**Utility:** This approach is recommended when there is insufficient confidence in the predictive value of the biomarker and that the novel treatment is believed to be effective in all individuals (i.e., the rationale for the biomarker is weak). This design can be used in order to avoid the possibility of missing an important treatment effect in the biomarker-positive patients (with insufficient benefit in the biomarker-negative subgroup). 

**Methodology:** The sample size should be set in such a way so as to yield adequate power for the overall test at the reduced significance level $a_{1}$ and for the potential biomarker positive subgroup analysis at significance level $a-a_{1}$ [60]. The fall-back version is identical to the parallel version of biomarker-positive and overall strategies in terms of sample sizes and study outcomes, however the difference between these approaches is that the fall-back strategy is useful in settings where a biomarker will be assessed only if the overall population benefit is not promising [14]. This strategy can test the treatment effectiveness in biomarker-positive patients even if there is no detected benefit of the novel treatment in the overall population. However, it does not evaluate clearly the treatment benefit in the biomarker-negative subpopulation.

**Statistical considerations:** As the two aforementioned biomarker-positive and overall designs, this strategy can again control the overall type I error α but it cannot control the probability of rejecting the null hypothesis of no treatment effect in the biomarker-negative subgroup when the treatment benefit is restricted to biomarker-positive patients. Consequently, there is high risk of inappropriately recommending the novel treatment for biomarker-negative patients. Song et al., 2007 [129] and George, 2008 [1] have discussed refinement of the significance levels associated with this design, which takes into account the correlation between the test for overall treatment effect and the test for the biomarker-positive treatment effect [60]. Additionally, a recent paper by Choai et al., 2015 [97] proposes a bias-corrected estimation method for treatment effects for the all-comers randomized clinical trials with a predictive biomarker which incorporate the fall-back analysis. For Choai et al., 2015 [97] the terminology “all-comers randomized clinical trials” is referred to the “Biomarker-positive and overall strategies with fall-back analysis”. More precisely, as this study design has an adaptive nature and is composed of two stages, a bias is possible to arise in the treatment effect estimation in the biomarker-positive subset when the first stage of the trial yields an overall result which is not significant and thus fails to demonstrate a treatment efficacy in the entire population. For this reason, Choai et al. ,2015 [97], formulate a bias function using polynomials in order to take into account the possibility of failing to demonstrate overall treatment efficacy during the first stage of the trial.

**Marker Sequential test design (MaST):** This design was identified in four papers (4%) of our review and while controlling the appropriate type I error rates, it evaluates not only the biomarker-positive and biomarker-negative subgroups but also the entire population sequentially to limit the assessment of treatment effect in the overall population when it seems that the biomarker-positive subgroup does not benefit from the novel treatment. A graphical illustration of this approach is given in Figure 10.

**Design:** In this design which owns an adaptive nature, first, the biomarker-positive subgroup is tested at a reduced level $a_{1}$ in [0,a] and if the result is significant, then the biomarker-negative subgroup is tested at the global significance level α. Otherwise, if the result is not significant, then the overall population is tested at level $a_{2}=a-a_{1\;}$ in order to make a treatment recommendation for the biomarker-negative patients.

**Utility:** It is generally recommended when robust evidence is available regarding a biomarker and there is prior evidence showing that the novel treatment is more beneficial for the biomarker-positive patients as compared to the biomarker-negative patients. Additionally, it is appropriate when we can assume that the treatment will not be beneficial for the biomarker-negative subgroup unless it is effective for the biomarker-positive subgroup. Additionally, the marker sequential test design is considered as an alternative to the sequential subgroup-specific design when the aim is to consider the treatment effect not only in biomarker-positive but also in the biomarker-negative patients.

**Methodology:** Freidlin et al., 2014 [69] recommended using the value of 0.022 for the reduced significance level $a_{1}$ in order to control the type I error rate for biomarker-negative patients at the global significance level α=0.025 and the value of 0.04 for the reduced significance level $a_{1}$ in order to control the type I error rate for biomarker-negative patients at the global significance level α=0.05.

Regarding the sample size for such a design where there is prior evidence indicating strong predictive ability of the biomarker, a standard sample size calculation (i.e., the same sample size calculation as for the enrichment designs) can be used for biomarker-positive subpopulation or alternatively, researchers can use the sample size calculation used for the sequential subgroup-specific design. However, in order to have sufficient number of biomarker-positive patients to detect treatment effectiveness in that particular biomarker-defined subset and consequently to reach the desired power, the sample size should be calculated using the reduced level $a_{1}$ [0,a] instead of the global significance level α which is used in the sample size formulae of the enrichment and sequential subgroup-specific designs. This will result in a small increase in the number of patients as compared to the enrichment and sequential subgroup-specific designs. Otherwise, if the reduced significance level $a_{1}$ is not used, this would yield minor loss of power.

**Statistical consideration:** Freidlin et al., 2014 [69] performed a comparison between the MaST and the sequential subgroup-specific design through a simulation study and concluded that the marker sequential design yields higher power in cases where the treatment effect is homogeneous across biomarker-defined subgroups. Additionally, with this approach, the power is preserved in situations where the experimental treatment is effective only for the biomarker-positive patients. Furthermore, in situations where biomarker status is not available for a portion of patients included in the trial, the marker sequential test design can either exclude these patients or include them in the global test, whereas, the proposed subgroup-specific designs do not consider inclusion of these patients in the analyses. If researchers decide to exclude patients with unavailable biomarker status from the study when using a MaST design, no statistical adjustment is required. On the other hand, if the inclusion of this study population is chosen, then this can result in inflation of the type I error rate for the biomarker-negative subpopulation above the global significance level α due to the modification of correlation structure between the biomarker-defined subgroup tests and global test. In addition, while both MaST and subgroup-specific designs have the ability to control the probability of incorrectly rejecting the null hypothesis of no treatment effect in the biomarker-negative patients at the significance level α when the experimental treatment does not work in either biomarker-defined subgroup, the sequential subgroup-specific approach typically has a smaller probability of incorrectly rejecting the null hypothesis of no treatment effect in the biomarker-negative subset (when the null hypothesis is true) as compared to the MaST design, especially under the global null hypothesis of no treatment effect in the entire population; the probability of incorrectly rejecting the null hypothesis of no treatment effect in the biomarker-negative patients depends on the choice of $a_{1}$. This conservativeness of sequential subgroup-specific design, which is due to its sequential nature, makes the MaST design advantageous [69].

#### 2.3.2. Hybrid Designs

Hybrid designs (Phase III) were identified in 14 papers (14%) of our review and they can be included in the all-comers designs, where the entire population is firstly screened for biomarker status and all individuals enter the trial. A graphical illustration of this design is given in Figure 11.

**Design:** In this approach, only the biomarker-positive patients are randomly assigned to either the experimental treatment group or to the control treatment group whereas the biomarker-negative patients receive the control treatment. These designs were first defined by Mandrekar and Sargent [30,31]. The difference compared with the enrichment designs is that the biomarker-negative patients are not excluded from the study. 

**Utility:** Hybrid designs can be used when there is compelling prior evidence which shows detrimental effect of the experimental treatment for a specific biomarker-defined subgroup (i.e., biomarker-negative subgroup) or some indication of its possible excessive toxicity in that subgroup, thus making it unethical to randomize the patients within this population to the experimental treatment.

**Methodology:** Similar to the enrichment design, hybrid designs are powered to identify treatment effect only in the biomarker-defined subgroup which is randomly assigned to the experimental or control treatment groups. Consequently, the same formula used for the required number of patients or events for the enrichment designs can be used for hybrid designs. This design is a combination of an enrichment design where we randomize patients to either the experimental or the control treatment group and a single-arm design in biomarker-negative patients.

**Statistical considerations:** The strength of the hybrid design is that apart from the evaluation of the predictive ability of a biomarker, the feasibility of a prognostic biomarker can also be tested. It can be considered as an advantageous design of the enrichment designs when there is prior evidence showing not only that the control treatment works well for the biomarker-negative population but also a detrimental effect of the experimental treatment for that subgroup or possible excessive toxicity as we do not exclude these patients from the trial as it happens in the enrichment designs.

### 2.4. Biomarker-Strategy Designs

Generally, with biomarker-strategy designs, the study population is randomized to treatment strategies as opposed to treatments per se. More precisely, patients are randomized to either a biomarker-based treatment strategy arm where the biomarker is used in deciding on approach to treatment, or to an arm that does not use the biomarker to guide treatment. Consequently, biomarker-strategy designs make a comparison between two strategies—one which uses biomarker information to inform treatment approach and the other that does not.

These designs are also known as biomarker-based strategy designs or signature-based strategy designs and they are composed of four subtypes; (i) biomarker-strategy designs with biomarker assessment in the control arm; (ii) biomarker-strategy designs without biomarker assessment in the control arm; (iii) biomarker-strategy designs with treatment randomization in the control arm and (iv) reverse marker-based strategy designs. Whilst patients randomized to the non-biomarker based strategy arm in the first two design subtypes are allocated the control treatment, in the third design subtype those patients undergo secondary randomization to either the control or experimental treatment. The fourth design subtype differs from the three aforementioned subtype designs as the non-biomarker based strategy arm is replaced by the reverse marker-strategy arm. The first and second types are similar with the difference being only in terms of ethical/feasibility issues regarding the acquisition of biomarker status at the beginning of the trial.

This approach is preferred when the study is planned for a confirmatory phase of a certain biomarker-based strategy allowing for comparison between the biomarker-based strategy and non-biomarker-based strategy.

#### 2.4.1. Biomarker-Strategy Design with Biomarker Assessment in the Control Arm

This approach is described in 21 (21%) papers of our review. 

**Design:** First, the study population enrolled in the trial is tested for its marker status. Next, patients irrespective of their biomarker status are randomized either to the biomarker-based strategy arm (also referred to as personalized arm) or to the non-biomarker-based strategy arm. In the biomarker-based strategy arm, biomarker-positive patients receive the experimental treatment, whereas, biomarker-negative patients receive the control treatment. Patients who are randomized to the non-biomarker-based strategy arm receive the control treatment irrespective of their biomarker status. A graphical illustration of this design is given in Figure 12. This biomarker-strategy design can be extended to more than one experimental treatment. More precisely, this extension is referred to as Individual profile design in literature and was identified in two papers [36,72] (2%) of our review. This design includes different individual status, e.g., instead of biomarker-positive and biomarker-negative subgroups we can have patients who are positive for biomarker 1, biomarker 2, biomarker n, leading to the selection of personalized treatments, (patients who are positive for biomarker 1 are treated with the corresponding experimental treatment 1, etc.).

**Utility:** This approach is useful when we want to test the hypothesis that the treatment effect based on the biomarker-based strategy approach is superior to that of the standard of care.

**Methodology:** The clinical utility of a biomarker can be evaluated by comparing the two strategy groups. The predictive utility of the marker-based treatment strategy could be assessed by comparing the outcome of all patients in the biomarker-based strategy arm to all patients in the non-biomarker-based strategy arm. Patients in the marker-based strategy arm do not need to be limited to two treatments; in principle, a marker-based strategy involving many biomarkers and many possible treatments could be compared to standard of care treatment.

According to Freidlin et al., 2010 [61], assuming a survival outcome, the required sample size in terms of number of events for this type of biomarker-strategy design in order to reach power (1−β) at significance level α (type I error) can be given by

(32) $$D_{strategy\;I}=4\;{\left[\frac{\left(z_{\alpha /2}+z_{\beta }\right)}{k\;{\log\theta }_{1}}\right]}^{2},$$

where k denotes the prevalence of biomarker-positive patients, θ<1 denotes the assumed hazard ratio in the biomarker-positive subpopulation and $z_{\alpha /2},\;z_{\beta }$ denote the upper α/2- and upper β-points respectively of a standard normal distribution where α and β denote the assumed type I error and type II error respectively. According to Freidlin et al. 2010 [61], it is assumed that there is no treatment effect in the biomarker-negative subpopulation (corresponding to a hazard ratio of experimental treatment versus control treatment of 1) and that there is no prognostic effect of the biomarker under the control treatment. Consequently, the overall hazard ratio between experimental and control arms in biomarker-positive patients and biomarker-negative patients can be approximated by $\exp\left[k\;\log\theta +\left(1-k\right)\;\log1\right]=\theta^{k}$ [61] and this is the reason why the formula which gives the required total number of events $\left(D_{strategy}\right)$ contains only the hazard ratio of biomarker-positive patients. Freidlin et al., 2010 [61] provided the aforementioned formula assuming that all random assignments use 1:1 randomization.

Additionally, Young et al., 2010 [26] determined the total sample size needed for this type of biomarker-strategy designs when using continuous clinical endpoints by

(33) $$N_{strategy\;I}=\frac{2{\left(z_{1-\alpha /2}+z_{1-\beta }\right)}^{2}\left(\tau_{m}^{2}+\tau_{n}^{2}\right)}{{\left(v_{m}-v_{n}\right)}^{2}},$$

where $z_{1-\alpha /2}$, $z_{1-\beta }$ denote the lower 1−α/2- and lower 1−β-points respectively of a standard normal distribution, α and β denote the assumed type I error and type II error respectively, $v_{m}$ and $v_{n}$ denote the mean response from the biomarker-based strategy arm and the non-biomarker-based strategy arm respectively, and $\tau_{m}^{2},\;\tau_{n}^{2}$ denote the variance of response for the biomarker-based strategy arm and non-biomarker-based strategy arm respectively. Young et al., 2010 [26] also provided formulae for the aforementioned variances which depend on sensitivity and specificity of the assay, such that any error in the evaluation of biomarker in the biomarker-based strategy can be accounted for.

For the case of binary outcomes, Eng, 2014 [92] provided the formula for the required sample size for each arm in a test of proportions between the two randomization arms (biomarker-based strategy arm and non-biomarker-based strategy arm). This formula can be given by

(34) $$N_{strategy\;I/arm}=\frac{{\left(z_{a}+z_{1-\beta }\right)}^{2}\left[g_{1}\left(1-g_{1}\right)+g_{2}\left(1-g_{2}\right)\right]}{\Delta_{2}^{2}}$$

where α corresponds to the target level, 1−β corresponds to the power, $g_{1}$ is the expected response rate in the biomarker-based strategy arm, $g_{2}$ is the expected response rate in the non-biomarker-based strategy arm and $\Delta_{2}=g_{1}-g_{2}$. The expected response rates $g_{1},g_{2}$ can be found by calculating the formulae $kr_{A+}+\left(1-k\right)r_{B-}$ and $r_{B}$ respectively, the prevalence of biomarker-positive patients corresponds to k and $r_{A+},r_{B-}$ are the assumed response rates of biomarker-positive patients receiving the experimental treatment and biomarker-negative patients receiving the control treatment, $r_{B}$ denotes the marginal effect of treatment B (control treatment).

**Statistical considerations:** This type of designs is able to inform researchers whether the biomarker is prognostic, since both biomarker positive and negative patients are exposed to the control treatment, but it cannot answer the question of whether the biomarker is predictive since only biomarker positive patients are exposed to the experimental treatment. Additionally, these designs have been criticized by many authors as less efficient than the marker-stratified designs since it is possible for some patients in both the biomarker-based strategy arm and non-biomarker-based strategy arm to be assigned to the same treatment (due to the existence of biomarker-negative patients in both strategy arms the treatment effect can be diluted) and they require a large sample size to detect an overall difference in outcomes between arms. Furthermore, these designs cannot compare experimental treatment to control treatment directly as they are designed to compare not the treatments but the biomarker-strategies. Another limitation of these designs is the uncertainty about whether the results which indicate efficacy of the biomarker-directed approach to treatment are caused due to a true effect of the biomarker or due to a treatment effect irrespective of the biomarker status.

#### 2.4.2. Biomarker-Strategy Design without Biomarker Assessment in the Control Arm

This strategy was identified in 14 papers (14%) of our review.

**Design:** In this approach, patients are again randomized between testing strategies (i.e., biomarker-based strategy and non-biomarker-based strategy) but it differs in terms of the timing of biomarker evaluation. More precisely, first, patients are randomized to either the biomarker-based strategy or to the non-biomarker-based strategy. Next, this design evaluates the biomarkers only in patients who are assigned to the biomarker-based strategy. Patients who are found to be biomarker-positive will receive the experimental treatment and patients who are biomarker-negative will receive the control treatment. On the other hand, the population which is randomized to the non-biomarker-based strategy will receive the control treatment. A graphical illustration of this design is given in Figure 13.

**Utility:** This design is useful in situations where it is either not feasible or ethical to test the biomarker in the entire population due to several logistical (e.g., specimens not submitted), technical (e.g., assay failure) or clinical reasons (e.g., tumor inaccessible); thus the biomarker status is obtained only in patients who are tailored to the biomarker-based strategy arm.

**Methodology:** The same mathematical formula for sample size calculation assuming a continuous clinical outcome proposed by Young et al. (2010) [26] and the formula assuming binary outcome proposed by Eng, 2014 [92] for the biomarker-strategy design with biomarker assessment in the control arm could be applied. Further, in terms of survival outcome, the same formula provided for the required number of events in the first version of biomarker-strategy designs (i.e., biomarker-strategy design with biomarker assessment in the control arm) could be considered.

**Statistical considerations:** These designs have the same advantages and limitations as the previously discussed biomarker-strategy design with biomarker assessment in the control arm, e.g., they have been criticized for their lack of efficiency due to the fact that biomarker negative patients are exposed to the control treatment in both arms of the trial. An additional limitation is that the biomarker-positive and biomarker-negative subpopulations might be more imbalanced as compared with the first type of biomarker-strategy design due to the fact that the randomization is performed before the evaluation of biomarker (balancing the randomization is useful to ensure that all randomized patients have tissue available).

#### 2.4.3. Biomarker-Strategy Design with Treatment Randomization in the Control Arm

Sargent and Allegra [108] proposed another version of Biomarker-strategy designs where there is a second randomization between experimental and control treatment in the non-biomarker guided strategy arm. This strategy is referred to in 17 papers (17%) of our review.

**Design:** A graphical illustration of this approach is given in Figure 14. The two previously described biomarker-strategy designs can answer the question about whether the biomarker-based strategy is more effective than standard treatment, irrespective of the biomarker status of the study population, whereas the biomarker-strategy design with treatment randomization in the control treatment is able to inform us about whether the biomarker-based strategy is better than not only the standard treatment but also better than the experimental treatment in the overall population. This is achieved by using a second randomization the ratio of which should be informed by the prevalence of the biomarker in question in the population as a whole to ensure balance between the study arms. Patients are first randomly assigned to either the biomarker-based strategy arm or to the non-biomarker-based strategy arm. Next, patients who are allocated to the non-biomarker-based strategy are again randomized either to the experimental treatment arm or to the standard treatment arm irrespective of their biomarker status. Patients who are allocated to the biomarker-based strategy and who are biomarker-positive are given the experimental treatment and patients who are biomarker-negative are given the control treatment. The clinical utility of the biomarker is evaluated by comparing treatment effect between the biomarker-based strategy arm and non-biomarker-based strategy arm. Such an approach can also identify whether a novel treatment is more effective in the entire population or in a biomarker-defined subgroup only, since both biomarker subgroups are exposed to both treatments.

**Utility:** These designs are preferable as compared to the two previously discussed biomarker-strategy designs in cases where there is interest in whether the biomarker is not only prognostic but also predictive.

**Methodology:** Mandrekar and Sargent, 2009 [31] calculated the total required sample size in terms of number of events for the comparison of a survival outcome in the biomarker-based strategy versus the non-biomarker-based strategy. According to them, the required total number of events when using 1:1 randomization to treatment arms is given by

(35) $$D_{strategy\;III}=\frac{4{\left(z_{a/2}+z_{\beta }\right)}^{2}}{{\left\{\log\left[\frac{2km_{B+}\;+\;2\left(1-k\right)m_{A-}}{k\left(m_{A+}\;+\;m_{B+}\right)\;+\;\left(1-k\right)\left(m_{A-}\;+\;m_{B-}\right)}\right]\right\}}^{2}},$$

where κ denotes the prevalence of the biomarker-positive patients, $m_{A+},m_{A-},m_{B+},m_{B-}$, denote the median survival for biomarker-positive and biomarker-negative patients receiving control and experimental treatments respectively. Also, the constants $z_{\alpha /2},\;z_{\beta }$ denote the upper α/2- and upper β-points respectively of a standard normal distribution where α and β denote the assumed type I error and type II error respectively.

Additionally, Young et al., 2010 [26], considering continuous clinical outcomes, calculated the total sample size by

(36) $$N_{strategy\;III}=\frac{2{\left(z_{1-\alpha /2}+z_{1-\beta }\right)}^{2}\left(\tau_{m}^{2}+\tau_{nr}^{2}\right)}{{\left(v_{m}-v_{nr}\right)}^{2}},$$

where $z_{1-\alpha /2}$, $z_{1-\beta }$ denote the lower 1−α/2- and lower 1−β-points respectively of a standard normal distribution, α and β denote the assumed type I error and type II error respectively, $v_{m}$ and $v_{nr}$ denote the mean response from the biomarker-based strategy arm and the non-biomarker-based strategy arm,) and $\tau_{m}^{2},\;\tau_{nr}^{2}$ denote the variance of response for the biomarker-based strategy arm and non-biomarker-based strategy arm respectively. The only differences in the mathematical formula for the total sample size $n_{t}$ between this type of biomarker-strategy design and the first and second types mentioned above are the values of $v_{nr}$ and $\tau_{nr}^{2}$, to reflect the fact that in the non-biomarker-based strategy arm patients are randomly assigned to either the experimental or control treatment. Again, the formulae can be adjusted to account for uncertainty in biomarker assessment.

For the case of binary outcomes, Eng, 2014 [92] provided the formula for the required sample size for each arm in a test of proportions between the two randomization arms (biomarker-based strategy arm and non-biomarker-based strategy arm). This formula can be given by

(37) $$N_{strategy\;III/arm}=\frac{{\left(z_{a}+z_{1-\beta }\right)}^{2}\left[g_{1}\left(1-g_{1}\right)+g_{3}\left(1-g_{3}\right)\right]}{\Delta_{3}^{2}}$$

where α correspond to the target level, 1−β corresponds to the power, $g_{1}$ is the expected response rate in the biomarker-based strategy arm, $g_{3}$ is the expected response rate in the non biomarker-based strategy arm and $\Delta_{3}=g_{1}-g_{3}$. The expected response rates $g_{1},\;g_{3}$ can be found by calculating the formulae $kr_{A+}+\left(1-k\right)r_{B-}$ and $r_{A}/2+r_{B}/2$ respectively, $r_{A}$ and $r_{B}$ denote the marginal effect of treatment A (experimental treatment) and treatment B (control treatment) respectively. $r_{A+},\;r_{B-}$ are the assumed response rates of biomarker-positive patients receiving the experimental treatment and biomarker-negative patients receiving the control treatment. The prevalence of biomarker-positive patients corresponds to k.

**Statistical considerations:** Similar to both aforementioned biomarker-strategy designs, the biomarker-strategy design with treatment randomization in the control arm will need larger sample size as compared to the marker-stratified designs. However, one strength is that they allow clarification of whether the results which indicate efficacy of the biomarker-directed approach to treatment are caused due to a true effect of the biomarker or due to a treatment effect irrespective of the biomarker status which does not happen in the first two types of biomarker-strategy designs.

#### 2.4.4. Reverse Marker-Based Strategy Design

Eng, 2014 [92] proposed another version of biomarker-strategy designs where the non-biomarker-based strategy arm which is included in the three aforementioned subtypes of biomarker-strategy designs is replaced by the reverse marker-strategy arm. This strategy is referred to in four papers (4%) of our review.

**Design:** A graphical illustration of this approach is given in Figure 15. In this design patients are randomized either to the biomarker-based strategy arm or the reverse biomarker-based strategy arm. As in the previous three biomarker-strategy subtype designs, patients who are allocated to the biomarker-strategy arm receive the experimental treatment if they are biomarker-positive whereas biomarker-negative patients receive the control treatment. By contrast, patients who are randomly assigned to the reverse biomarker-based strategy arm receive control treatment if they are biomarker-positive, whereas biomarker-negative patients receive experimental treatment.

**Utility:** Reverse marker-based strategy is a more efficient strategy as compared to the first and third biomarker-strategy subtype design for testing the interaction hypothesis of treatment and biomarker. This design should be used in cases where prior evidence indicates that both experimental and control treatment are effective in treating patients but the optimal strategy has not yet been identified.

**Methodology:** This subtype design is balanced (i.e., the randomization frequencies for each treatment are equal independent of the prevalence of the biomarker) and it is powered to evaluate the interaction between treatment and biomarker. For the case of binary outcomes, Eng, 2014 [92] provided the formula for the required sample size for each arm in a test of proportions between the two randomization arms (biomarker-based strategy arm and reverse biomarker-based strategy arm). This formula can be given by

(38) $$N_{strategy\;IV/arm}=\frac{{\left(z_{a}+z_{1-\beta }\right)}^{2}\left[g_{1}\left(1-g_{1}\right)+g_{4}\left(1-g_{4}\right)\right]}{\Delta_{4}^{2}}$$

where α correspond to the target level, 1−β corresponds to the power, $g_{1}$ is the expected response rate in the biomarker-based strategy arm, $g_{4}$ is the expected response rate in the reverse biomarker-based strategy arm and $\Delta_{4}=g_{1}-g_{4}$. The expected response rates $g_{1},\;g_{4}$ can be found by calculating the formulae $kr_{A+}+\left(1-k\right)r_{B-}$ and $kr_{B+}+\left(1-k\right)r_{A-}$ respectively, $r_{A+},\;r_{B-}$ are the assumed response rates of biomarker-positive patients receiving the experimental treatment and biomarker-negative patients receiving the control treatment and $r_{A-},\;r_{B+}$ are the assumed response rates of biomarker-negative patients receiving the experimental treatment and biomarker-positive patients receiving the control treatment The prevalence of biomarker-positive patients corresponds to k.

**Statistical considerations:** This design enables the evaluation of the interaction between the biomarker and different treatments and can estimate directly the marker-strategy response rate. Additionally, this subtype design allows the estimation of the effect size of the experimental treatment compared to the control treatment for each biomarker-defined subgroup separately. Also, there is no chance that the same treatment will be tailored to biomarker-positive patients who are randomized either to the biomarker-based strategy arm or the reverse marker strategy (i.e., biomarker-positive patients in the biomarker-based strategy will be given only the experimental treatment and biomarker-positive patients in the reverse marker strategy arm will be given only the control treatment). Also, there is no possibility of the same treatment assignment to biomarker-negative patients who are randomly assigned to the two biomarker-based strategy arms (i.e., biomarker-negative patients in the marker-based strategy arm will be treated with the control treatment, whereas biomarker-negative patients in the reverse marker strategy arm will be treated with the experimental treatment). According to Eng, 2014 [92] who compared the reverse marker-based strategy design with the first (i.e., biomarker-strategy design with biomarker assessment in the control arm) and third (i.e., biomarker-strategy design with treatment randomization in the control arm) subtype of biomarker-strategy designs in the case of binary outcomes, the effect size in order to make a comparison of the different treatment strategy arms would be larger than in the first and third subtype designs. Furthermore, it has been shown by Eng, 2014 that in situations where a randomly chosen treatment has a better than 7% response rate, the reverse marker-based strategy design works better as compared to the third biomarker-strategy subtype (i.e., Biomarker-strategy design with treatment randomization in the control arm). It has also been demonstrated that this novel design is more than four times more efficient in order to test the interaction between treatment and biomarker compared to Biomarker-strategy design with biomarker assessment in the control arm, Biomarker-strategy design with randomization in the control arm and the marker stratified design. Eng, 2014 demonstrated the benefits of the Reverse Marker-Based strategy design with the aim to assess the interaction between treatment and biomarker. However, Baker, 2014 [93] stated that other designs than the Reverse Marker-Based strategy design would be more appropriate in order to investigate questions which include treatment effect of biomarker-defined subgroups and biomarker-based strategy arms.

### 2.5. Other Designs

#### A Randomized Phase II Trial Design with Biomarker Proposed by Freidlin et al., 2012

Freidlin et al., 2012 [71] proposed a biomarker-guided Phase II clinical trial design in which when it is completed, it recommends which type of Phase III trial should be used. These recommendations for a Phase III trial are the following: (i) enrichment design; (ii) marker-stratified design; (iii) a traditional trial design without a biomarker; or (iv) drop consideration of the experimental treatment. A graphical illustration of this design is given in Figure 16.

**Design:** For this type of randomized Phase II trial, it is assumed that the experimental treatment will be more beneficial among biomarker-positive patients than biomarker-negative patients without ruling out the efficacy of the novel treatment in biomarker-negative patients. The intermediate endpoint of progression-free survival (PFS) is used which is able not only to give the results earlier but also to target larger treatment effects as compared to overall survival (OS) endpoint. 

The design starts by comparing the experimental treatment with the control treatment in the biomarker-positive subgroup using a one-sided level of significance $a_{1}=0.10$. The null hypothesis is that the progression-free survival for biomarker-positive patients is the same for both experimental and control treatment arm ($HR_{0,\;biom+}\leq 1\;\mathrm{vs}.\;HR_{1,\;biom+}>1)$. Next, if the null hypothesis is rejected, which means that the experimental treatment is better than the control treatment in the biomarker-positive subgroup we continue with the calculation of an 80% two-sided confidence interval (CI) for the hazard ratio (control vs experimental) in the biomarker-negative subpopulation. Three decisions are made according to the values of the CI: (i) if the entire CI is less than 1.3 then we can continue with a Phase III enrichment design; (ii) if the CI includes the values 1.3 or 1.5 then we can continue with a Phase III marker-stratified design and (iii) if the entire CI is greater than 1.5 then it seems that the biomarker is not useful as the novel treatment benefits only the biomarker-negative patients, thus, the biomarker should be dropped and a traditional randomized Phase III design should be conducted. Otherwise, if the null hypothesis is not rejected at the one-sided significance $a_{1}=0.10$ (meaning that that the experimental treatment is not better than the experimental treatment in the biomarker-positive subgroup), then we continue with the comparison of treatments in the overall study population at one-sided level of significance a=0.05. If the null hypothesis of no treatment effect in the entire population is rejected, then the authors recommend to drop the biomarker and to continue with a traditional randomized Phase III trial due to the fact that the biomarker seems to be useless. On the other hand, if the null hypothesis is not rejected, the experimental treatment should not be tested further as it does not seem to be effective. 

**Utility:** This design should be used when we want to conduct a Phase II randomized trial which allows decisions to be made about which type of Phase III biomarker-guided trial to proceed with. It is appropriate when there is prior evidence that the novel treatment benefits mostly the biomarker-positive patients without ruling out treatment effect in biomarker-negative patients. 

**Methodology:** Freidlin et al., 2012 [71] have provided an online tool for calculating the sample size which can be found on the following website http://brb.nci.nih.gov/Data/FreidlinB/RP2BM [116]. In order for a sample size to be estimated, the following information is required: (i) the significance levels for testing the treatment effect in the biomarker-positive subgroup and in the entire population; (ii) cut-offs and confidence intervals for the hazard ratio in the biomarker-negative subgroup; (iii) the prevalence of biomarker-positive patients; (iv) the median progression-free survival in each treatment arm in each biomarker-defined subgroup and (v) the accrual parameters. Regarding the accrual parameters, the author specifies the minimum sample size for biomarker-positive patients for which the accrual continues until this number is reached, the maximum number of over-accrual in biomarker-positive subgroup for which the accrual to the entire population stops after this number is reached and the maximum accrual number in biomarker-negative patients for which the accrual to this biomarker-defined subgroup stops when this number is reached.

**Statistical considerations:** In real life, it might not be possible to obtain the biomarker status for the entire population. If the biomarker status is unknown for some patients, then these individuals could be included in the analysis of the overall population. More precisely, in case that the proportions of patients with unknown biomarker status is low, the randomization of them to either the experimental or the control treatment could be considered in the second stage of this Phase II trial where we test the treatment effectiveness in the entire population. Another statistical consideration is that researchers should take into account the adjustment for inflation in Phase III type I error as the chosen Phase III trial design depends on the performance of the aforementioned randomized Phase II trial. Additionally, the authors suggest generally that in cases where it seems that the control treatment has been shown more beneficial, an aggressive interim inefficacy/futility should be used, i.e., when the estimated hazard ratio of control treatment versus the experimental treatment is equal or less than one when half of the required number of events have been observed, then the accrual should stop to that biomarker-defined subgroup.

## 3. Discussion

A number of biomarker-guided trial designs have been proposed in the past decade, including both biomarker-guided adaptive and non-adaptive trial designs. We have undertaken a comprehensive review of the literature using an in-depth search strategy to report on the biomarker-guided designs proposed to date, with a view to providing the research community with clarity in definition, methodology and terminology of the various trial designs. The review is split in two parts due to its size; the first part of the review is focused on adaptive designs which are extensively discussed in our published paper “Biomarker-Guided Adaptive Trial Designs in Phase II and Phase III: a Methodological Review”, Antoniou et al., 2016 [35], whereas, herein we focus on non-adaptive designs which incorporate biomarkers. 

The review has demonstrated ambiguity and confusion regarding the biomarker-guided non-adaptive designs proposed by different authors. In this review, we focus on 5 main types of such designs including their subtypes and variations. Knowledge on how to implement and analyse these designs are essential in testing the effectiveness of a biomarker-guided approach to treatment; hence, a comprehensive review giving this knowledge is essential for the research community. In our in-depth study, we provide researchers with analytical information of these study designs not only in terms of their utility, advantages and limitations but also in terms of their methodology. In addition, a graphical illustration for each biomarker-guided design is given. A guidance document by Tajik et al., 2012 [117] regarding the evaluation of putative biomarkers in randomized clinical trials came to our knowledge by personal communication as we were not able to identify it during our literature search.

The non-adaptive designs do not allow modifications of important aspects of the trial such as refinement of the existing study population, treatment assignment, study endpoints, study duration, etc. In non-adaptive designs, all these factors are defined before the initiation of the study and they are kept fixed during the course of the clinical trial. However, there is a great potential of failure when implementing such conventional designs due to potential wrong design assumptions of the key aspects of the study that might be made before the conduct of the trial. Hence, an adaptive design clinical study which allows on-going adaptations based on accumulating study data from interim analysis might hold advantageous position as compared to the non-adaptive trial design due to its flexibility. However, before implementing an adaptive design a lot of issues should be taken into careful consideration by research teams in order to prove that there are good reasons for conducting such designs. Regulatory and logistical issues, requirement of additional efforts for the achievement of the design, potential difficulties, possible increased cost and time, statistical challenges including the potential increase of the chance of a false conclusion that the treatment is effective (inflation of Type I error) and whether the adaptation process has led to positive study results that are difficult to interpret irrespective of having control of Type I error should be considered [130]. A recent paper by Dimairo et al., 2015 [131] refers to a number of obstacles and barriers when implementing adaptive designs in practice. Several key stakeholders in clinical trials research have been interviewed (i.e., UK Clinical Trials Units directors, funding board and panel members, statisticians, regulators, chief investigators, data monitoring committee members and health economists) expressing difficulties of adaptive designs. Lack of appropriate knowledge and familiarity of these designs in the scientific community, insufficient time and funding structure, additional work required due to the complexity of such designs and the needed statistical expertise and appropriate software are some of the highlighted difficulties mentioned in the paper of Dimairo et al., 2015 [131]. In addition, this study includes the characterisation of potential benefits of an adaptive design to patients, clinical trials as well as funders.

The different designs proposed so far for biomarker-guided designs, both non-adaptive designs which remain an appealing approach to a great extent mainly due to their simplicity and adaptive designs which are more flexible need to be further explored by the research community, as the proper choice and use of such designs can result in a great increase in the efficiency of a trial and expedite the development of novel treatments.

The characteristics and methodology of the five main designs and their subtypes are discussed in the current paper, whilst information on their variations are summarized in File S1-S4. Additional references for these variations and the literature review search strategy are provided in [132,133].

## Figures and Tables

**Table 1:** Types of Biomarker guided non-adaptive designs proposed within the last ten years.

Types of Biomarker-Guided Non-Adaptive Trial Designs	Utility	Advantages	Limitations
**Single arm designs** (7 papers) [30,36,37,38,39,40,41] (see Figure 2)	Useful for initial identification and/or validation of a biomarker.	(**A1**) Considered as a simple statistical design as there is no need for randomization of patients.	(**L1**) There is no distinction between prognostic and predictive biomarker as patients are not randomized to experimental and control treatment arms.
**Also called:** Nonrandomized clinical trial design, Uncontrolled Cohort Pharmacogenetic Study design		(**A2**) Simple logistics.	
**Examples of actual trials:** None identified ^a^		(**A3**) Not complex statistical design	
		(**A4**) In some cases, these designs may be viewed as ethical as all patients are given the opportunity to experience the experimental treatment. However, they may be viewed as unethical if the novel treatment does not benefit a subgroup of patients or causes adverse events.	
**Enrichment designs** (71 papers) [1,4,7,8,9,11,13,15,16,18,19,21,23,25,26,27,28,29,30,31,32,33,36,42,43,44,45,46,47,48,49,50,51,52,53,54,55,56,57,58,59,60,61,62,63,64,65,66,67,68,69,70,71,72,73,74,75,76,77,78,79,80,81,82,83,84,85,86] (see Figure 3)	Useful when we aim to test the treatment effect only in biomarker-positive subset for which there is prior evidence that the novel treatment is beneficial, but the candidate biomarker requires prospective validation.	(**A5**) Evaluates the effect of the experimental treatment in the biomarker-positive subgroup in a simple and efficient way.	(**L2**) Do not assess whether the experimental treatment benefits the biomarker-negative patients, thus we cannot obtain information about this subgroup. Also unable to demonstrate whether the targeted treatment is beneficial in the entire study population.
**Also called:** Targeted design, Selection design, Efficient Targeted design, Biomarker-Enrichment design, Marker-enrichment design, Gene enrichment design, Enriched design, Clinically enriched Phase III study design, Clinically Enriched Trial design, Biomarker-Enriched design, Biomarker Enriched design, Biomarker Selected trial design, Screening enrichment design, Randomized Controlled Trial (RCT) of test positive design, Population enrichment design	Useful when it is not ethical to assign biomarker-negative patients to the novel treatment for which there is prior evidence that it will not be beneficial for this subpopulation, or that it will harm them.	(**A6**) Provides clear information about whether the novel treatment is effective for the biomarker-positive subgroup, thus these designs can identify the best treatment for these patients and confirm the usefulness of the biomarker.	(**L3**) Do not inform us directly about whether the biomarker is itself predictive because the relative treatment efficacy may be the same in the unevaluated biomarker-negative patients. Since these designs only enrol a subgroup of patients, they do not allow for full validation of the marker’s predictive ability. For full validation, a trial would need to randomize all patients in order to test for a treatment–biomarker interaction.
**Examples of actual trials:** CRYSTAL [49], BRIM 3 [49,50,51], EURTAC [49], CLEOPATRA [49], PROFILE 1007 [49,50], LUX-Lung [49], NSABP B-31 and NCCTG N9831 [4,15,16,18,19,28,29,30,31,36,44,46,52,53,54,55,56,57,58,59,60], CALGB-10603 [61], CATNON [62], CODEL [62], Evaluation of epidermal growth factor receptor variant III (EGFRvIII) peptide vaccination [62], N0923 [7,21] , Flex study [64], TOGA trial [47], IPASS [33,43], N0147 [29], PetaCC-8 [29,47], C80405 [29], ECOG E5202 [29]	Recommended when both the cut-off point for determination of biomarker-status of patients and the analytical validity of a biomarker are well established.	(**A7**) Reduced sample size as the assessment of treatment effect is restricted only to biomarker-positive subgroup. Therefore, if the selected biomarker is “biologically correct” and reliably measured, the used enrichment strategy could result in a large saving of randomized patients.	(**L4**) Researchers should carefully decide whether or not to follow this strategy as it may be of limited value due to the exclusion of biomarker-negative patients. It may be that the entire population could benefit from the experimental treatment equally irrespective of biomarker status, in which case enrolling only the biomarker-positive patients will result in slow trial accrual, increase of expenses and unnecessary limitation of the size of the indicated patient population.
		(**A8**) Enables rapid accumulation of efficacy data.	(**L5**) Concern over an ethical problem as we cannot include individuals in a clinical trial if it is believed that the treatment is not effective for them, as raised by the US Food and Drug Administration (FDA) [50]. It was based on the facts that the experimental treatment can only be approved for a particular biomarker-defined subpopulation (i.e., biomarker-positive patients) if a companion diagnostic test is also approved, and how the test can be approved if the Phase III trial does not show that the novel treatment does not benefit the biomarker-negative patients.
		(**A9**) Allow us to avoid potential dilution of the results due to the absence of biomarker-negative patients. For example, if the design had included the biomarker-negative population and the biomarker positivity rate was low as compared to the biomarker negative rate, then the estimation of the overall treatment effectiveness could be diluted as it would be driven by the biomarker-negative subset.	(**L6**) The accuracy of diagnostic devices used to identify the biomarkers, e.g., biomarker assays, is not always correct [45]. This can result in incorrect selection of biomarker-positive patients and therefore these patients will erroneously be enrolled in a trial yielding biased treatment effect estimates. For example, even when the experimental treatment works well for a specific subgroup, if the biomarker assay is not able to identify this subgroup robustly then a promising treatment may be abandoned.
		(**A10**) Can be attractive in terms of speed and cost, meaning that patients are provided with tailored treatment sooner.	
**Marker Stratified designs** (45 papers) [4,10,12,13,15,16,17,18,19,21,25,26,27,30,31,33,44,45,46,49,50,51,53,58,61,62,66,68,71,72,73,74,79,80,81,84,85,86,87,88,89,90,91,92,93] (see Figure 4)	Useful when there is evidence that the novel treatment is more effective in the positive biomarker-defined subgroup than in the negative biomarker-defined subgroup but there is insufficient compelling data indicating that the experimental treatment does not benefit the biomarker-negative patients.	(**A11**) Ability to assess the treatment effect not only in the entire population but also in each biomarker-defined subgroup. Thus, this design can find the optimal treatment in the entire population and in each biomarker-defined subgroup.	(**L7**) In situations where there are several biomarkers and treatments this design may not be feasible as it involves randomization of patients between all possible treatment options and may require a large sample size.
**Also called:** Marker-stratified design, Biomarker-stratified design, Stratified-Randomized design, Stratification design, Stratified design, Stratified Analysis design, Marker by treatment – interaction design, Marker-by-treatment interaction design, Treatment by marker interaction design, Treatment-by-marker interaction design, Marker × treatment interaction design, Treatment-marker interaction design, Biomarker-by-treatment interaction design, Non-targeted RCT (stratified by marker) design, Genomic Signature stratified designs, Signature-Stratified design, Randomization or analysis stratified by biomarker status design, marker-interaction design.		(**A12**) An ethical design even in situations where the biomarker is not useful as no treatment decisions are made based on biomarker status; all decisions are made randomly. Consequently, if the biomarker’s value is in doubt, this design may be preferred.	(**L8**) May not be feasible when the prevalence of the biomarker is low.
**Examples of actual trials:** MARVEL (N023) [4,16,30,31,33,44,61,89], GALGB-30506 [15,61], RTOG0825 [45], EORTC 10994 p53 [12,66], IBCSG trial IX [18], MINDACT [18]			(**L9**) Might be expensive to test the entire population for its biomarker status.
			(**L10**) Measuring the biomarker up front may be logistically difficult.
			(**L11**) There is no guarantee of balanced groups for analysis.
**Sequential Subgroup-Specific design** (11 papers) [13,14,19,22,53,57,58,60,69,91,94] (see Figure 5)	Recommended when prior evidence indicates that the biomarker-positive subpopulation benefits more from the novel treatment as compared to the biomarker-negative subpopulation.	(**A13**) Allows for the estimation of treatment effect in biomarker-positive and biomarker-negative subgroups.	(**L12**) Has less power when there is homogeneity of treatment across the different biomarker defined subgroups as compared to the overall/biomarker-positive designs.
**Also called:** sequential design, Fixed-sequence 2 design, hierarchical fixed sequence testing procedure		(**A14**) Preserves the overall type I error rates and allows for a smaller sample size than the parallel version mentioned below.	(**L13**) Need a much larger sample size than the overall/biomarker positive designs if we assume that the treatment effect is relatively homogeneous across the biomarker-defined subsets.
**Examples of actual trials:** PRIME [49], MARVEL [49]		(**A15**) Considered as the best direct evidence for clinical decision making as it tests the treatment effectiveness in both the biomarker-positive and biomarker-negative subset in a sequential way.	
		(**A16**) Do not require larger sample size than the overall/biomarker-positive designs when the prevalence of the biomarker-positive patients is small.	
**Parallel Subgroup-Specific design** (3 papers) [14,49,69] (see Figure 6)	Appropriate when the aim of the study is to give treatment recommendations for each biomarker-defined subgroup separately at the same time.	(**A17**) Same as (**A13**), (**A16**)	(**L14**) Same as (**L12**)
**Also called:** Phase III Biomarker-Stratified design			(**L15**) Allocates the overall level a between the two biomarker-defined subgroup tests which means that it will be more difficult to achieve statistical significance in the biomarker-positive subgroup.
**Examples of actual trials:** None identified ^a^			
**Biomarker-positive and overall strategies with parallel assessment** (8 papers) [1,14,36,47,49,69,95,96] (see Figure 7)	Recommended when the aim of the study is to assess the treatment effect in both the entire population and in the biomarker-positive subset but not in the biomarker-negative population.	(**A18**) Can control the overall type I error a.	(**L16**) Can be overly conservative as in the SATURN trial because of the correlation between the test of treatment effect in the overall study population and in the biomarker subgroups.
**Also called:** Overall/biomarker-positive design with parallel assessment, prospective subset design, hybrid design		(**A19**) Can require smaller sample size as compared to the subgroup-specific designs, especially when we assume that the novel treatment equally benefits both biomarker-defined subgroups.	(**L17**) Cannot control the probability of rejecting the null hypothesis of no treatment effect in the biomarker-negative subset when the treatment benefit is restricted to biomarker-positive patients. Consequently, there is a high risk of inappropriately recommending the novel treatment for biomarker-negative patients due to the large treatment effect in biomarker-positive subset.
**Examples of actual trials:** S0819 [14,49], SATURN [14,36,47,49,95,96], MONET1 [14,49], ARCHER [14,49], ZODIAC [49], MERiDiAN [49]			
**Biomarker-positive and overall strategies with sequential assessment** (11 papers) [13,14,30,44,49,69,80,84,85,88,94] (see Figure 8)	Might be useful in cases where the experimental treatment is expected to be effective in the overall population.	(**A20**) Same as (**A18**), (**A19**)	(**L18**) Can be problematic for determining whether the treatment is beneficial in the biomarker-negative subgroup.
**Also called:** Overall/biomarker-positive design with sequential assessment, sequential design, Fixed-sequence 2 design, hierarchical fixed sequence testing procedure			(**L19**) Same as (**L17**)
**Examples of actual trials:** Trial of letrozole plus lapatinib versus letrozole plus placebo in breast cancer, with the biomarker defined by human epidermal growth factor receptor 2 (HER2) [14], N0147 [30,49]			
**Biomarker-positive and overall strategies with fall-back analysis** (15 papers) [10,30,36,44,47,49,53,57,60,69,84,88,94,96,97] (see Figure 9)	Recommended when there is insufficient confidence in the predictive value of the biomarker and the novel treatment is assumed to probably benefit all patients.	(**A21**) Can assess the treatment effect in the biomarker-positive patients, if no benefit is detected in the overall population.	(**L20**) Same as (**L17**), (**L18**)
**Also called:** Biomarker-stratified design with fall-back analysis, fall-back design, prospective subset design, sequential design, other analysis plan design, Fallback design		(**A22**) Same as (**A18**), (**A19**)	
**Examples of actual trials:** None identified ^a^			
**Marker Sequential test design** (4 papers) [14,49,69,94] (see Figure 10)	Recommended when biomarkers with strong credentials are available and we have convincing evidence that the novel treatment is more effective in biomarker-positive than in biomarker-negative patients.	(**A23**) Can provide clear evidence of treatment benefit in the biomarker-positive subgroup and in the biomarker-negative subgroup.	(**L21**) In situations where biomarker status is not available for some of the patients included in the study, this design can either exclude these patients or include them in the global test, however, further statistical adjustments might be required in that case.
**Also called:** MaST design, hybrid design	Appropriate when we can assume that the treatment will not be beneficial in the biomarker-negative subpopulation unless it is effective for the biomarker-positive subpopulation.	(**A24**) Enables sequential testing of the treatment effect in the entire study population and in the biomarker-defined subgroups to restrict testing of the treatment effect in the entire population when there is no significant result in the biomarker-positive subset, while controlling the appropriate type I error rates.	(**L22**) Does not decrease the sample size of the study as it was developed in order to increase the power compared to the sequential subgroup-specific design in situations where the novel treatment benefits equally both biomarker-negative and biomarker-positive patients.
**Examples of actual trials:** ECOG E1910 [14,49]		(**A25**) Results in higher power as compared to the sequential subgroup-specific design in cases where the treatment effect is homogeneous across the biomarker-defined subgroups.	
		(**A26**) Preserves the power in situations where the treatment effect is restricted only to the biomarker-positive patients and at the same time it controls the relevant type I error rates.	
		(**A27**) Control the type I error rate for the biomarker-negative subgroup over all possible prevalence values.	
		(**A28**) The probability of erroneously concluding that the novel treatment is beneficial for the entire population when the global effect is driven by the biomarker-positive patients is minimized since the design only tests the treatment effect in the entire population when no significant effect is detected in the biomarker-positive subgroup.	
**Hybrid designs** (14 papers) [1,13,15,29,30,31,36,46,48,55,66,84,88,98] (see Figure 11)	Can be used when there is prior evidence indicating that only a particular treatment is beneficial to a biomarker-defined subgroup which makes it unethical to randomize patients with that specific biomarker status to other treatment options.	(**A29**) The feasibility of a prognostic biomarker can be tested.	None found.
**Also called:** Mixture design, Combination of trial designs, hybrid biomarker design		(**A30**) Allows for better risk assessment and improved individualized treatment since it assigns patients to treatments based on risk assessment scores instead of their biomarker status (biomarker-positive and biomarker-negative patients).	
**Examples of actual trials:** TAILORx [15,48,55,58,63,66], EORTC MINDACT [15,48,55,66], ECOG 5202 study [30,46]			
**Biomarker-strategy designs with biomarker assessment in the control arm** (21 papers) [15,25,26,32,33,36,45,61,62,64,79,82,85,86,92,93,99,100,101,102,103] (see Figure 12)	Useful when we want to test the hypothesis that the treatment effect based on the personalized approach is superior to that of the standard of care.	(**A31**) Biomarker can be validated without including all possible biomarker–treatment combinations [26] as in the non-biomarker-based arm all patients receive only the control treatment.	(**L23**) Unable to inform us whether the biomarker is predictive as these designs are able to answer the question about whether the biomarker-based strategy is more effective than standard treatment, irrespective of the biomarker status of the study population.
**Also called:** Marker strategy design, Biomarker-strategy design, Strategy design, Marker-based strategy design, Marker-based design, Random disclosure design, Customized strategy design, Parallel controlled pharmacogenetic study design, Marker-based strategy design I, Biomarker-guided design, Biomarker-based assignment of specific drug therapy design, Marker-based strategy I design, Biomarker-strategy design with a standard control, Marker strategy design for prognostic biomarkers		(**A32**) Have the option of testing the biomarker status of patients in the non-biomarker-strategy arm which can aid secondary analyses [26].	(**L24**) The evaluation of the true biomarker by treatment effect is not possible as the biomarker-positive patients receive only the experimental treatment and not the alternative treatment (control treatment). Consequently, this design cannot detect the case in which the control treatment might be more beneficial for the entire population.
**Examples of actual trials:** GILT docetaxel [15], Randomized phase III trial conducted in Spain, dedicated to patients with advanced Non-Small Cell Lung Cancer (NSCLC) candidates for first-line chemotherapy [32,64,100], Study the effect of Magnetic Resonance Imaging (MRI) in patients with low back pain on patient outcome and to evaluate Doppler US of the umbilical artery in the management of women with intrauterine growth retardation (IUGR), Randomized controlled trial in recurrent platinum-resistant ovarian carcinoma [101]		(**A33**) Able to inform us whether the biomarker is prognostic.	(**L25**) In case that the number of biomarker-positive patients is very small, then the treatment received will be similar in biomarker-strategy arm and non-biomarker strategy arm. Consequently, the trial might give little information regarding the efficacy of the experimental treatment or it might not be able to detect it. As a result, this type of design should be used when there is an adequate number of biomarker-positive and biomarker-negative patients.
		(**A34**) Can be expanded to investigate several biomarkers and treatments [103]. Additionally, these designs can be attractive when evaluating multiple biomarkers or the predictive value of molecular profiling between several treatment options is to be assessed [45].	(**L26**) Unable to compare directly experimental treatment to control treatment as the aim is to compare not the treatments but the biomarker-strategies.
		(**A35**) Might be used more frequently in the future due to the wide variety of molecular biomarkers, complexity of gene expression arrays, and several treatments directed at similar targets [103].	(**L27**) Less efficient designs than biomarker-stratified designs [4,73] and a poor substitute for clinical trials which aim to compare the experimental treatment to control treatment, since it is possible for some patients in both the biomarker-based strategy arm and non-biomarker-based strategy arm to be assigned to the same treatment (due to the existence of biomarker-negative patients in both strategy arms the treatment effect can be diluted) [51]. Consequently, as a large overlap of patients receiving the same treatment might have occurred, the comparison of the two biomarker-strategy arms results in a hazard ratio which is forced towards unity, i.e., no treatment effect exists as the effect of experimental versus control treatment is diluted by the biomarker-based treatment selection. For this reason, a large sample size is needed to detect at least a small overall difference in outcomes between the two biomarker-strategy arms.
			(**L28**) Should be used only if you want to evaluate a complex biomarker-guided strategy with a variety of treatment options or biomarker categories [73].
**Biomarker-strategy design without biomarker assessment in the control arm** (14 papers) [9,13,17,18,20,25,36,38,61,74,101,104,105,106] (see Figure 13)	In situations where it is not feasible or unethical to test the biomarker in the entire population.	(**A36**) Galanis et al., 2011 [45] stated that these designs can be attractive when evaluating multiple biomarkers or the predictive value of molecular profiling between several treatment options is to be assessed. Also, Freidlin and Korn, 2010 [73] claimed that these biomarker-strategy designs should be used only if researchers want to evaluate a complex biomarker-guided strategy with a variety of treatment options or biomarker categories.	(**L29**) Criticized for their potential cost increase due to the fact that patients without predicted responsive biomarker are double enrolled in the trial (biomarker-negative patients receive control treatment in both strategy arms).
**Also called:** Biomarker-strategy design with standard control, Direct-predictive biomarker-based, RCT of testing, Test-treatment, Parallel controlled pharmacogenetic diagnostic study, Marker strategy, Marker-based with no randomization in the non-marker-based arm, Classical, Marker-based strategy, Marker strategy design for prognostic biomarkers		(**A37**) Same as (**A31**), (**A32**), (**A33**)	(**L30**) Biomarker-positive and biomarker-negative subpopulations might be more imbalanced as compared with the first type of biomarker-strategy design due to the fact that the randomization to different treatment strategies is performed before the evaluation of the biomarker status (balancing the randomization is useful to ensure that all randomized patients have tissue available). This can happen especially when the number of patients is very small.
**Examples of actual trials:** A study, which evaluated the use of immediate computed tomography in patients with acute mild head injury [101,104].			(**L31**) Same as (**L23**), (**L24**), (**L25**), (**L26**), (**L27**)
**Biomarker-strategy design with treatment randomization in the control arm** (17 papers) [15,17,26,27,32,36,45,62,64,66,74,86,92,93,106,107,108] (see Figure 14)	In cases where we want to know whether the biomarker is not only prognostic but also predictive, these designs are preferable as compared to the two previously mentioned biomarker-strategy designs.	(**A38**) These designs have the ability to inform researchers about the potential superiority of the control treatment in the whole population or among a particular biomarker-defined subpopulation.	(**L32**) Generally require a larger sample size as compared to the marker-stratified designs.
**Also called:** Biomarker-strategy design with a randomized control, Modified marker-based strategy design (for predictive biomarkers), Biomarker-strategy design with randomized control, Marker-based design with randomization in the non-marker-based arm, Marker-based strategy design II, Marker-strategy design, Augmented strategy design, Trial design allowing the evaluation of both the treatment and the marker effect		(**A39**) Able to inform us whether the biomarker is prognostic or predictive.	(**L33**) Same as (**L27**)
**Examples of actual trials:** None identified ^a^		(**A40**) Allow clarification of whether the results which indicate efficacy of the biomarker-directed approach to treatment are caused due to a true effect of the biomarker status or to an improved treatment irrespective of the biomarker status.	
		(**A41**) Same as (**A36**)	
**Reverse marker-based strategy** (4 papers) [86,92,93,109] (see Figure 15)	Enables testing the interaction hypothesis of treatment and biomarker in a more efficient way as compared to the first (i.e., Biomarker-strategy design with biomarker assessment in the control arm) and third biomarker-strategy subtype design (i.e., Biomarker-strategy design with randomization in the control arm and the marker stratified design)	(**A42**) Can estimate directly the marker-strategy response rate.	(**L34**) It has been claimed by Baker, 2014 [93] that other designs than the reverse marker-based strategy are more appropriate in order to investigate questions which include both treatment effect of biomarker-defined subgroups and the biomarker strategy treatment effect. These designs should allow the estimation of treatment effects within biomarker-defined subgroups as well as the estimation of the global treatment effect.
**Also called:** None found		(**A43**) Allows the estimation of the effect size of the experimental treatment compared to the control treatment for each biomarker-defined subset separately.	
**Examples of actual trials:** None identified ^a^		(**A44**) There is no chance that the same treatment will be tailored to biomarker-positive patients who are randomized either to the biomarker-based strategy arm or the reverse marker strategy. Also, there is no possibility of the same treatment assignment to biomarker-negative patients who are randomly assigned to the two biomarker-based strategy arms.	
		(**A45**) It has been demonstrated by Eng, 2014 [92] that this new type of design is more than four times more efficient for testing the interaction between treatment and biomarker compared to Biomarker-strategy design with biomarker assessment in the control arm, Biomarker-strategy design with randomization in the control arm and the marker stratified design.	
**A specific randomized phase II trial design that can be used to guide decision making for further development of an experimental therapy.** (1 paper) [71] (see Figure 16)	Recommended when we want to conduct a Phase II randomized trial which allows decisions to be made about which type of Phase III biomarker-guided trial should be used.	(**A46**) Works well in providing recommendations for phase III trial design.	None found

^a^ Although not found within the review, the design may be implemented in ongoing trials.

**Table 2:** Sample size formulae for biomarker-guided clinical trial designs.

Types of Biomarker-Guided Non-Adaptive Trial Designs	Sample Size Formula	Definition
**Single arm designs**	Standard sample size formula can be used, more information can be found in the ‘methodology’ part of the ‘Single arm designs’ section in the main text.	
**Enrichment designs** [55,61,65,110,111,112]	Online tool for sample size calculation when using either binary or time-to-event endpoints is available on the following website: http://brb.nci.nih.gov/brb/samplesize/td.html [113].	
	$E\left(D_{i,enrichment}\right)=\frac{nT\lambda_{i}}{2\left(\lambda_{i}+\varphi_{i}\right)}\left\{1-\frac{e^{-\left(\lambda_{i}+\varphi_{i}\right)\tau }}{\left(\lambda_{i}+\varphi_{i}\right)T}\left[1-e^{-\left(\lambda_{i}+\varphi_{i}\right)T}\right]\right\}$	$E\left(D_{i,enrichment}\right)$ is referred to the expected number of events per treatment arm (time-to-event outcome), i corresponds to either the experimental or the control treatment group, 1:1 ratio between the two treatment arms (experimental:control) is assumed, λ corresponds to the event hazard rate, φ is the loss to follow-up rate, T denotes the accrual time, patients enter the trial according to a Poisson process with rate n per year over the accrual period of T years, *τ* corresponds to the follow-up period.
	$D_{enrichment}=4{\left[\frac{\left(z_{\alpha /2}+z_{\beta }\right)}{{\log\theta }_{1}}\right]}^{2}$	$D_{enrichment}$ is referred to the required total number of events (time-to-event outcome), 1:1 ratio between the two treatment arms (experimental:control) is assumed, $z_{\alpha /2},\;z_{\beta }$ denote the upper α/2- and upper β-points respectively of a standard normal distribution, α and β denote the assumed type I error and type II error respectively, $\theta_{1}$ denotes the assumed hazard ratio between the two treatment groups (control vs experimental) in the biomarker-positive subset.
	$N_{enrichment/arm}=2{\bar{p}}_{Q}\left(1-{\bar{p}}_{Q}\right){\left[\frac{\left(z_{\alpha /2}+z_{\beta }\right)}{\left(p_{A}^{Q}-p_{B}\right)}\right]}^{2}$	$N_{enrichment/arm}$ is referred to the required number of patients per treatment arm (binary outcome), 1:1 ratio between the two treatment arms (experimental:control) is assumed, $p_{A}^{Q}$ and $p_{B}$ are the response probabilities in the experimental and control groups respectively, ${\bar{p}}_{Q}=\left(p_{A}^{Q}+p_{B}\right)/2$.
	$N_{enrichment/arm}=\frac{2\sigma^{2}{\left(z_{\alpha /2}+z_{\beta }\right)}^{2}}{{\left(\mu_{A+}-\mu_{B+}\right)}^{2}}$	$N_{enrichment/arm}$ is referred to the required total number of patients per treatment arm (continuous response endpoints), 1:1 ratio between the two treatment arms (experimental:control) is assumed, $\sigma^{2}$ denotes the anticipated common variance, $\mu_{A+}$ and $\mu_{B+}$ the mean responses for biomarker-positive patients in the experimental and control treatment arm respectively.
	$N_{enrichment/arm}=2\sigma^{2}{\left(z_{\alpha /2}+z_{\beta }\right)}^{2}{\left\{\lambda_{1}\left[\left(1-\omega \right)\;\zeta +\omega \right]\right\}}^{-2}$	$N_{enrichment/arm}$ is referred to the required total number of patients per treatment arm (continuous response endpoints when accounting for error in the assaying of the study population), 1:1 ratio between the two treatment arms (experimental:control) is assumed, ω measures the accuracy of the assay and corresponds to the PPV (positive predictive value of the assay, i.e., the proportion of patients who are assigned biomarker positive status according to the assay who are truly biomarker positive), $\lambda_{1}$ is the treatment effect in the biomarker-positive patients and $\zeta =\lambda_{0}/\lambda_{1}$ (where $\lambda_{0}$ is the treatment effect in the biomarker-negative patients).
**Marker Stratified designs** [31,53,60,92,111,112,114]	Online tool for sample size calculation when using either binary or time-to-event endpoints is available on the following website: http://brb.nci.nih.gov/brb/samplesize/sdpap.html [115].	
	$D_{stratified}=4\frac{{\left(z_{a_{1}}+z_{\beta }\right)}^{2}}{{\left[\log\left(\theta_{1}\right)\right]}^{2}}+4\frac{{\left(z_{a_{2}}+z_{\beta }\right)}^{2}}{{\left[\log\left(\theta_{2}\right)\right]}^{2}}$	$D_{stratified}$ is referred to the required total number of events for the achievement of sufficient power in each biomarker-defined subgroup separately (time-to-event endpoint), 1:1 ratio between the two treatment arms (experimental:control) is assumed, $\theta_{2}$ corresponds to the hazard ratio of biomarker-negative subgroup, $a_{1}=a_{2}=a/2$.
	$D_{stratified}=\frac{4{\left(z_{a/2}+z_{\beta }\right)}^{2}}{{\left[k\log\left(\theta_{1}\right)+\left(1-k\right)\log\left(\theta_{2}\right)\right]}^{2}}$	$D_{stratified}$ is referred to the required total number of events for the achievement of sufficient power in the overall population (time-to-event endpoint), k is the proportion biomarker-positive patients, 1:1 ratio between the two treatment arms (experimental:control) is assumed.
	$N_{stratified}=\frac{4{\left(z_{a/2}+z_{\beta }\right)}^{2}}{{\left\{\left[kPr_{\left(+\right)}\left(event\right)\log\left(\theta_{1}\right)+\left(1-k\right)Pr_{\left(-\right)}\left(event\right)\log\left(\theta_{2}\right)\right]/\sqrt{kPr_{\left(+\right)}\left(event\right)+\left(1-k\right)Pr_{\left(-\right)}\left(event\right)}\right\}}^{2}}$	$N_{stratified}$ is referred to the required total number of patients for the achievement of sufficient power in the overall population (time-to-event endpoint), 1:1 ratio between the two treatment arms (experimental:control) is assumed, $Pr_{\left(+\right)}\left(event\right)$, $Pr_{\left(-\right)}\left(event\right)$ are the probabilities of an event in biomarker-positive subset and biomarker-negative subset respectively.
	$\frac{D_{stratified}}{D_{enrichment}}=\frac{{\left[\log\left(\theta_{1}\right)\right]}^{2}}{{\left[k\log\left(\theta_{1}\right)+\left(1-k\right)\log\left(\theta_{2}\right)\right]}^{2}}=\frac{1}{{\left[k+\left(1-k\right)\frac{\log\left(\theta_{2}\right)}{\log\left(\theta_{1}\right)}\right]}^{2}}$	$\frac{D_{stratified}}{D_{enrichment}}$ is referred to the ratio of the required number of events between marker stratified and enrichment design (time-to-event endpoint).
	$\frac{N_{stratified}}{N_{enrichment}}\approx \frac{1}{{\left[k+\left(1-k\right)\frac{\delta_{-}}{\delta_{+}}\right]}^{2}}$	$\frac{N_{stratified}}{N_{enrichment}}$ is referred to the ratio of the required number of patients between marker stratified and enrichment design (binary outcome), $\delta_{-}$, $\delta_{+}$, correspond to the treatment effectiveness in biomarker-negative and biomarker-positive subgroup respectively.
	$N_{stratified}=2{\left(z_{a}+z_{1-\beta }\right)}^{2}\left\{\frac{r_{A+}\left(1-r_{A+}\right)+r_{B+}\left(1-r_{B+}\right)}{{\left(\beta_{A}+\beta_{I}\right)}^{2}}+\frac{r_{A-}\left(1-r_{A-}\right)+r_{B-}\left(1-r_{B-}\right)}{{\left(\beta_{A}\right)}^{2}}\right\}$	$N_{stratified}$ is referred to the required total number of patients (binary outcome), $\beta_{0}$ denotes a baseline effect, $\beta_{A}$ denotes the added effect of the experimental treatment, $\beta_{+}$ denotes the biomarker-positive effect and $\beta_{I}$ denotes the nonadditive effect, α corresponds to the target level, 1−β corresponds to the power, $r_{A+},\;r_{B+}$ are the assumed response rates of biomarker-positive patients receiving the experimental and the control treatment respectively, $r_{A-},\;r_{B-}$ are the assumed response rates of biomarker-negative patients receiving the experimental and the control treatment respectively.
**Sequential Subgroup-Specific design** [57]	$N_{sequential\;subgroup-specific}^{+}=N_{enrichment}$	$N_{sequential\;subgroup-specific}^{+}$ is referred to the required number of biomarker-positive patients (binary outcome), $N_{enrichment}$ is the required number of biomarker-positive patients (binary outcome) in the enrichment design.
	$N_{sequential\;subgroup-specific}=\frac{N_{enrichment}}{k}$	$N_{sequential\;subgroup-specific}$ is referred to the required total number of patients (binary outcome), $N_{enrichment}$ is the required number of biomarker-positive patients (binary outcome) in the enrichment design.
	$N_{sequential\;subgroup-specific}^{-}=\frac{\left(1-k\right)N_{enrichment}}{k}$	$N_{sequential\;subgroup-specific}^{-}$ is referred to the required number of biomarker-negative patients (binary outcome), $N_{enrichment}$ is the required number of biomarker-positive patients (binary outcome) in the enrichment design.
	$D_{sequential\;subgroup-specific}^{+}=D_{enrichment}$	$D_{sequential\;subgroup-specific}^{+}$ is referred to the required number of events for biomarker-positive patients (time-to-event outcome), $D_{enrichment}$ is the required number of events for biomarker-positive patients (time-to-event outcome).
	$D_{sequential\;subgroup-specific}^{-}=D_{enrichment}\left(\frac{\lambda_{-}}{\lambda_{+}}\right)\left(\frac{1-k}{k}\right)$	$D_{sequential\;subgroup-specific}^{-}$ is referred to the required number of events for biomarker-negative patients (time-to-event outcome), $D_{enrichment}$ is the required number of events for biomarker-positive patients (time-to-event outcome), $\lambda_{-}$, $\lambda_{+}$, are the event rates in biomarker-negative and biomarker-positive control subgroups.
**Parallel Subgroup-Specific design**	Same formula proposed for marker stratified designs could be considered to achieve sufficient power in each biomarker-defined subgroup simultaneously. However, in order to control the overall type I error rate of the design at the overall level of significance α it is required to allocate this overall α between the test for the biomarker-positive subgroup and the test for the biomarker-negative. Consequently, for biomarker-positive subgroup the reduced significance level $a_{1}=a-a_{2}$ can be used whereas the reduced significance level $a_{2}=a-a_{1}$ can be used for biomarker-negative subgroup.	
**Biomarker-positive and overall strategies with parallel assessment**	If there is significant confidence that the biomarker is predictive, the sample size estimation is aimed at having a sufficient number of biomarker-positive individuals to enable the treatment effect in the biomarker positive subgroup to be detected. Standard formula for sample size calculation of biomarker-positive subgroup proposed for the enrichment designs could be considered by using the reduced significance level $a_{1}=a-a_{2}$. On the other hand, if there is no confidence in the predictive value of the biomarker, the sample size estimation is aimed at having a sufficient number of patients to detect a treatment effect in the overall study population; consequently, for the sample size calculation, the same formula proposed for marker stratified designs aiming to achieve sufficient power in the overall population could be applied by using the reduced significance level $a_{2}=a-a_{1}$.	
**Biomarker-positive and overall strategies with sequential assessment**	At the first stage, the standard formula for a traditional randomized trial which is the same with the formula proposed for enrichment designs can be applied for the biomarker-positive subgroup. At the second stage, the sample size formula proposed for marker stratified designs aiming to yield appropriate power for the entire population could be considered.	
**Biomarker-positive and overall strategies with fall-back analysis**	At the first stage, the sample size formula proposed for marker stratified designs aiming to yield appropriate power for the entire population could be considered by using the reduced significance level $a_{1}=a-a_{2}$. At the second stage, the formula proposed for enrichment designs could be applied for the biomarker-positive subgroup by using the reduced significance level $a_{2}=a-a_{1}$.	
**Marker Sequential test design (MaST) **	A standard sample size calculation (i.e., the same sample size calculation as for the enrichment designs) can be applied for the biomarker-positive subpopulation. However, in order to have sufficient number of biomarker-positive patients to detect treatment effectiveness in that particular biomarker-defined subset and consequently to reach the desired power, the sample size should be calculated by using the reduced significance level $a_{1}$ [0,a] instead of the global significance level α which is used in the sample size formulae of the enrichment designs. The same formula could be considered for the sample size calculation of the biomarker-negative subgroup; however, the corresponding hazard ratio of that subgroup and the global significance level α should be used. For the sample size calculation of the entire population, the same formula proposed for marker stratified designs aiming to achieve sufficient power in the overall population could be considered by using the reduced significance level $a_{2}=a-a_{1}$.	
**Biomarker-strategy, design with biomarker assessment in the control arm** [26,61,92]	$D_{strategy\;I}=4{\left[\frac{\left(z_{\alpha /2}+z_{\beta }\right)}{k{\log\theta }_{1}}\right]}^{2}$	$D_{strategy\;I}$ is referred to the required total number of events (time-to-event outcome), 1:1 ratio between the two treatment arms (experimental:control) is assumed.
	$N_{strategy\;I}=\frac{2{\left(z_{1-\alpha /2}+z_{1-\beta }\right)}^{2}\left(\tau_{m}^{2}+\tau_{n}^{2}\right)}{{\left(v_{m}-v_{n}\right)}^{2}}$	$N_{strategy\;I}$ is referred to the required total sample size (continuous clinical endpoints), 1:1 ratio between the two treatment arms (experimental:control) is assumed, $z_{1-\alpha /2}$, $z_{1-\beta }$ denote the lower 1−α/2- and lower 1−β-points respectively of a standard normal distribution, $v_{m}$ and $v_{n}$ denote the mean response from the biomarker-based strategy arm and the non-biomarker-based strategy arm respectively, and $\tau_{m}^{2},\;\tau_{n}^{2}$ denote the variance of response for the biomarker-based strategy arm and non-biomarker-based strategy arm respectively.
	$N_{strategy\;I/arm}=\frac{{\left(z_{a}+z_{1-\beta }\right)}^{2}\left[g_{1}\left(1-g_{1}\right)+g_{2}\left(1-g_{2}\right)\right]}{\Delta_{2}^{2}}$	$N_{strategy\;I/arm}$ is referred to the required total number of patients per arm (binary outcome), $g_{1}$ is the expected response rate in the biomarker-based strategy arm, $g_{2}$ is the expected response rate in the non biomarker-based strategy arm, $\Delta_{2}=g_{1}-g_{2}$, $g_{1},g_{2}\;$can be found by calculating the formulae $kr_{A+}+\left(1-k\right)r_{B-}$ and $r_{B}$ respectively, $r_{B}$ denotes the marginal effect of treatment B (control treatment).
**Biomarker-strategy design without biomarker assessment in the control arm**	Same formulae as for the ‘Biomarker-strategy design with biomarker assessment in the control arm’ can be considered.	
**Biomarker-strategy design with treatment randomization in the control arm** [26,31,92]	$D_{strategy\;III}=\frac{4{\left(z_{a/2}+z_{\beta }\right)}^{2}}{{\left\{\log\left[\frac{2km_{B+}+2\left(1-k\right)m_{A-}}{k\left(m_{A+}+m_{B+}\right)+\left(1-k\right)\left(m_{A-}+m_{B-}\right)}\right]\right\}}^{2}}$	$D_{strategy\;III}$ is referred to the required total number of events (time-to-event outcome), 1:1 ratio between the two treatment arms (experimental:control) is assumed, $m_{A+},m_{A-},\;m_{B+},m_{B-}$, denote the median survival for biomarker-positive and biomarker-negative patients receiving control and experimental treatments respectively.
	$N_{strategy\;III}=\frac{2{\left(z_{1-\alpha /2}+z_{1-\beta }\right)}^{2}\left(\tau_{m}^{2}+\tau_{nr}^{2}\right)}{{\left(v_{m}-v_{nr}\right)}^{2}}$	$N_{strategy\;III}$ is referred to the required total sample size (continuous clinical endpoints), 1:1 ratio between the two treatment arms (experimental:control) is assumed, $v_{nr}$ denotes the mean response from the non-biomarker-based strategy arm, $\tau_{nr}^{2}$ denotes the variance of response for the non-biomarker-based strategy arm respectively.
	$N_{strategy\;III/arm}=\frac{{\left(z_{a}+z_{1-\beta }\right)}^{2}\left[g_{1}\left(1-g_{1}\right)+g_{3}\left(1-g_{3}\right)\right]}{\Delta_{3}^{2}}$	$N_{strategy\;III/arm}$ is referred to the required total number of patients per arm (binary outcome), $g_{3}$ is the expected response rate in the non biomarker-based strategy arm and $\Delta_{3}=g_{1}-g_{3}$, the expected response rate $g_{3}$ can be found by calculating the formula $r_{A}/2+r_{B}/2$, $r_{A}$ denotes the marginal effect of treatment A (experimental treatment).
**Reverse marker-based strategy** [92]	$N_{strategy\;IV/arm}=\frac{{\left(z_{a}+z_{1-\beta }\right)}^{2}\left[g_{1}\left(1-g_{1}\right)+g_{4}\left(1-g_{4}\right)\right]}{\Delta_{4}^{2}}$	$N_{strategy\;IV/arm}$ is referred to the required total number of patients per arm (binary outcome), $g_{4}$ is the expected response rate in the reverse biomarker-based strategy arm and $\Delta_{4}=g_{1}-g_{4}$, the expected response rate $g_{4}$ can be found by calculating the formula $kr_{B+}+\left(1-k\right)r_{A-}$, $r_{B+},\;r_{A-}$ are the assumed response rates of biomarker-positive patients receiving the control treatment and biomarker-negative patients receiving the experimental treatment.
**Randomized Phase II trial design with biomarkers** [71]	Online tool for sample size calculation is available on the following website: http://brb.nci.nih.gov/Data/FreidlinB/RP2BM [116].	

## Supplementary Materials

The following are available online at www.mdpi.com/2075-4426/7/1/1/s1, File S1-S4: Extensions of Biomarker-guided non-adaptive trial designs, Keywords S1: Literature review search strategies for both biomarker-guided clinical trial designs and for traditional trial designs.
