# Correction: Antoniou, M.; et al. Biomarker-Guided Non-Adaptive Trial Designs in Phase II and Phase III: A Methodological Review. *J. Pers. Med*. 2017, *7*, 1

**DOI:** 10.3390/jpm8020017

**Published:** 2018-05-07

**Authors:** Miranta Antoniou, Ruwanthi Kolamunnage-Dona, Andrea L. Jorgensen

**Affiliations:** 1 MRC North West Hub for Trials Methodology Research, Liverpool L69 3GL, UK; Ruwanthi.Kolamunnage-Dona@liverpool.ac.uk (R.K.-D.); A.L.Jorgensen@liverpool.ac.uk (A.L.J.); 2 Department of Biostatistics, Institute of Translational Medicine, University of Liverpool, Liverpool L69 3GL, UK

The authors wish to make the following corrections to this paper [1]:

On page 26, the sentence, “If the overall test is not significant, then the experimental treatment is compared to the control treatment in the biomarker-positive patients using the type I error *a* = 0.05.” should be “If the overall test is not significant, then the experimental treatment is compared to the control treatment in the biomarker-positive patients using the significance level 0.05 − *a*.”

They would also like to remove the last paragraph of page 32 due to inaccurate description of the adaptive signature design:

“The same analysis plan was used in the adaptive signature design which is further described in our methodological review regarding the biomarker-guided adaptive designs, Antoniou et al., 2016 [35]. More precisely, the difference between the adaptive signature design and the fall-back design is the following: in the adaptive signature design, in case that the first stage failures to show treatment effectiveness in the entire population, then the study population is divided in order to develop and validate a biomarker, using a split sample strategy, whereas in the biomarker-positive and overall strategies design with fall-back analysis the biomarker assessment is conducted at the beginning of the trial. However, both of the designs test at the first stage the entire population at the significance level *a*_1_ and at the second stage the biomarker-positive patients at the significance level *a*_2_ = *a* − *a*_1_.”

These changes have no material impact on the conclusions of the paper. The authors would like to apologize for any inconvenience caused to the readers by these changes.

## References

[1] Antoniou M., Kolamunnage-Dona R., Jorgensen A.L. (2017). Biomarker-Guided Non-Adaptive Trial Designs in Phase II and Phase III: A Methodological Review. J. Pers. Med..

