# Supplementary material for: Biomarker-Guided Non-Adaptive Trial Designs in Phase II and Phase III: A Methodological Review
Source: J Pers Med. 2017 Jan 25;7(1):1. doi: 10.3390/jpm7010001 (PMC5374391; doi:10.3390/jpm7010001)
Supplement: Supplementary file 1 [file jpm-07-00001-s001.pdf]

Supplementary Materials: Biomarker-Guided Non-Adaptive Trial Designs in Phase II and Phase III: A Methodological Review

Miranta Antoniou, Ruwanthi Kolamunnage-Dona and Andrea L. Jorgensen

File S1-S4. Extensions of Biomarker-Guided Non-Adaptive Trial Designs

Variations of Biomarker-Strategy Designs

Sequential before-after pharmacogenetic diagnostic study: The design identified in two papers [36,38] (2%) of our review. A graphical illustration of this design is given in Figure S1.

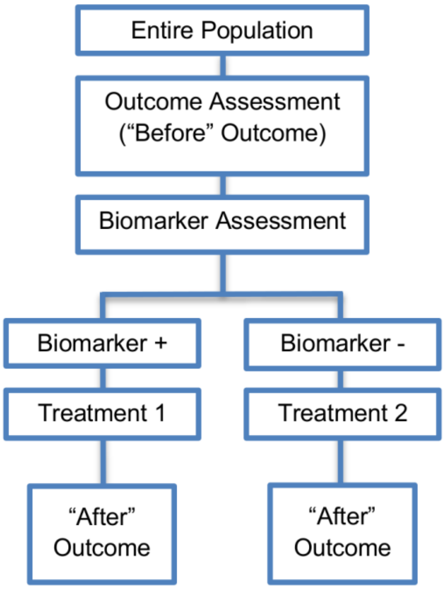

Figure S1. Sequential before–after pharmacogenetic diagnostic study.

This approach was proposed in the field of pharmacogenetics involving the assessment of pharmacogenetic diagnostics being performed during the study. In this sequential approach each patient serves as his/her own control) [38]. Treatments are tailored to patients before genotyping and then again after genotyping. A comparison of outcomes before and after the introduction of pharmacogenomics is conducted.

This individual crossover approach requires a smaller number of patients as compared to the previous designs described and is not considered complex in its implementation. Additionally, before–after comparisons are the basis of medical practice in many important therapeutic areas such as surgery [38] and they can inform researchers about whether a personalized treatment is more effective than the standard of care. However, types of systematic error (i.e., bias which yield incorrect estimate of a measure of disease) might be introduced, e.g., due to errors on classification of outcomes or on the assessment of the biomarker status of patients.

Classifier randomization design: Another extension of biomarker-strategy designs is the Classifier randomization design which was identified in two papers [36,107] (2%) of our review. An example of an actual trial which uses this strategy is the NNBC-3 European trial [107]. A graphical illustration of this design is given in Figure S2.

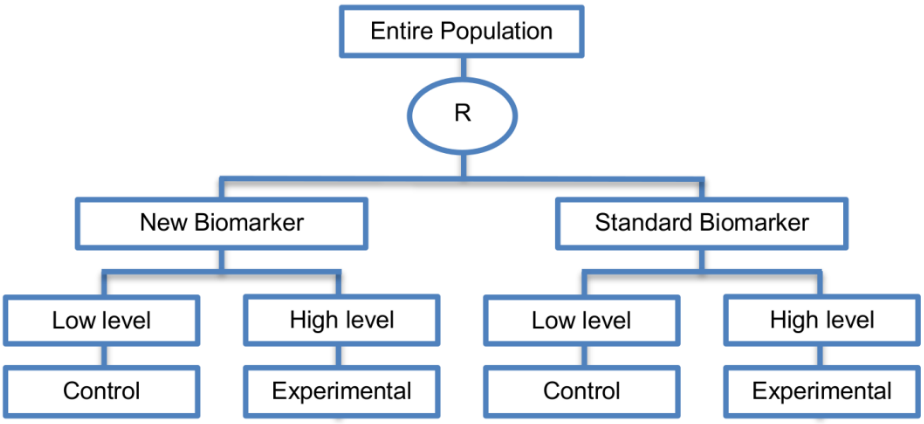

Figure S2. Classifier randomization design. "R" refers to randomization of patients.

This is an approach proposed for the validation of prognostic biomarkers which randomly assign the study population between classifiers (i.e., a new biomarker and a standard biomarker) rather than treatments validating directly the new biomarker. With this approach, we compare the new and standard classifiers in order (i) to show equivalence in outcome, i.e., the outcome of patients assigned to the new biomarker is not too different to that obtained from patients in the standard classifier independently of the low/high level in each category; (ii) to show superiority in outcome, i.e., the outcome of patients assigned to the new classifier who are given the experimental treatment is superior to that obtained from the patients given the control treatment.

Modified marker strategy design: This modified version of the biomarker-strategy design was identified in five papers [9,19,22,58,91] (5%) of our review. A graphical illustration of this approach is given in Figure S3.

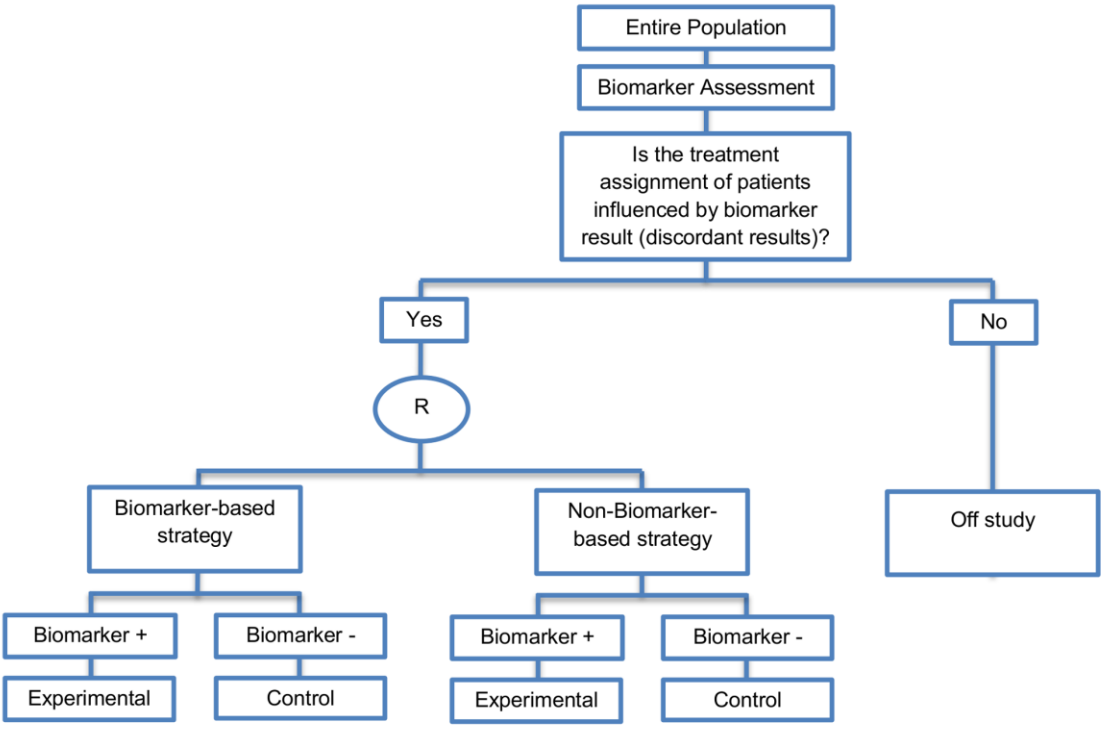

Figure S3. Modified marker strategy design. "R" refers to randomization of patients.

In this design, biomarker assessment is required in the entire population before randomization to either the biomarker-based strategy arm or to the non-biomarker-based strategy arm. However, only patients for whom treatment assignment would be influenced by biomarker result are then

randomized—those whose treatment assignment would be the same regardless of biomarker result are off study. An example of an actual trial which uses this approach is the MINDACT trial. More precisely, in the MINDACT trial the entire population is evaluated by two analyses, the 70-gene profile (biomarker-based analysis) as well as clinicopathologic factors (non-biomarker-based analysis). Next, only patients with discordant results (i.e., patients predicted to be high risk based on one of the two analyses and low risk patients based on the other analysis) will be randomized to either the biomarker-based strategy or to the non-biomarker-based strategy).

Next, as exactly in the biomarker-strategy design with biomarker assessment in the control arm, patients who are randomly assigned to the biomarker-based strategy arm are treated either with the experimental treatment if they have biomarker-positive status or with the control treatment if they have biomarker-negative status. The main limitation of biomarker-strategy designs is that they can result in a significant number of patients in the trial who are assigned to the same treatment in both biomarker-based strategy arm and non-biomarker-based strategy arm (i.e., biomarker-negative patients in the biomarker-based strategy arm receive control treatment but, biomarker-negative patients might also receive control treatment in the non-biomarker-based strategy arm as the random assignment of the entire population to this strategy arm is independent of their biomarker-status). Consequently, a large sample size is needed to identify the diluted treatment effect. This modified version promises to solve this limitation of biomarker-strategy designs by only randomizing patients for whom treatment assignment is influenced by biomarker result.

Variation of Randomize-All Designs

Two-way stratified design: A version of the Biomarker Stratified design is the Two-way Stratified design which was referred to in two papers [107,132] (2%) of our review. Figure S4 represents the graphical illustration of this strategy.

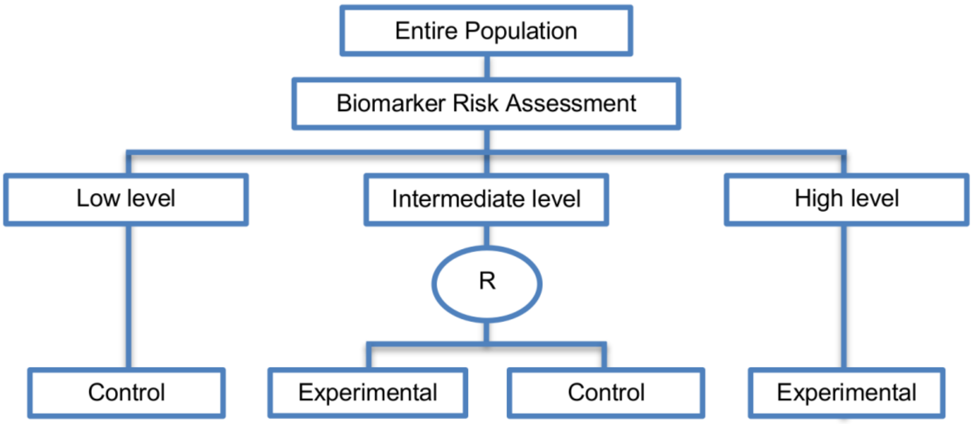

Figure S4. Two-way Stratified design (for validation of prognostic biomarkers). "R" refers to randomization of patients.

In this approach, patients are stratified according to their biomarker results. Standard treatment is tailored to patients with a low score, experimental treatment is given to patients with a high score and those with an intermediate score are randomized to either experimental or standard treatment. An example of an actual trial which uses this approach is the that conducted by the "Arbeitsgemeinschaft Gynakologische Onkologie" (AGO) Study Group in cooperation with the EORTC Receptor and Biomarker Study Group for the "Chemo-N0 trial" to validate UPA and PAI1 as prognostic indicators in node negative breast cancer [107].

According to Spira et al. [132] a noninferiority design for the intermediate group can be used and has the statistical power to detect a 3% or greater difference between the randomized arms.

Since there is no direct prospective comparison between the novel and standard classifier, the two-way stratified design provides further, although indirect, validation of the biomarker.

Keywords S1. Literature Review Search Strategies for Both Biomarker-Guided Clinical Trial Designs and for Traditional Trial Designs

MEDLINE

Traditional Clinical Trial Designs

1. Clinical Trials as Topic/
2. Clinical Trial/
3. 1 or 2
4. Research Design
5. design*.mp.
6. Research design*.mp.
7. Statistical design*.mp.
8. Study design*.mp.
9. Traditional design*.mp.
10. Trial design*.mp.
11. 4 or 5 or 6 or 7 or 8 or 9 or 10
12. 3 and 11
13. limit 12 to (english language and "review articles")
14. limit 13 to last ten years

MEDLINE

Biomarker-Guided Clinical Trial Designs

1. Clinical Trials as Topic/
2. clinical trial*. ti, ab.
3. 1 or 2
4. design*. ti, ab.
5. 3 and 4
6. limit 5 to comment
7. limit 5 to editorial
8. limit 5 to journal article
9. limit 5 to guideline
10. limit 5 to systematic reviews
11. limit 5 to "review"
12. limit 5 to technical report
13. limit 5 to practice guideline
14. 6 or 7 or 8 or 9 or 10 or 11 or 12 or 13
15. limit 14 to English language
16. limit 15 to last 10 years
17. exp *marker/or biological marker/or clinical marker/
18. (marker* or biomarker* or factor* or classifier or signature* or target* or endpoint). ti, ab.
19. 17 or 18
20. 16 and 19

The Ovid strategy was conducted by following the guidance by BMA Library—MEDLINE Plus. Basic Course. Notes for OvidSP; 2012. Available from: http://www.google.co.uk/url?sa=t&rct=j&q=&esrc=s&frm=1&source=web&cd=1&ved=0ahUKEwjS7_OmodvJAhWGVhQKHZr0AZMQFggdMAA&url=http%3A%2F%2Fbma.org.uk%2F-%2Fmedia%2Ffiles%2Fpdfs%2Fabout%2520the%2520bma%2Flibrary%2Fmedline%2520plus%2520basic%2520course%2520manual%252020 12.pdf&usg=AFQjCNGFxcWiS11CJsroeeIETAWjW0neUA [133].
